# Noninvasive identification of carbon-based black pigments with pump-probe microscopy

**DOI:** 10.1126/sciadv.adp0005

**Published:** 2024-12-11

**Authors:** Heidi V. Kastenholz, Michael I. Topper, Warren S. Warren, Martin C. Fischer, David Grass

**Affiliations:** ^1^ Department of Chemistry, Duke University, Durham, NC 27708, USA.; ^2^ Department of Physics, Duke University, Durham, NC 27708, USA.; ^3^ Department of Biomedical Engineering, Duke University, Durham, NC 27708, USA.; ^4^ Department of Radiology, Duke University, Durham, NC 27710, USA.

## Abstract

Carbon-based black pigments, a widely used class of pigments, are difficult to differentiate with the noninvasive techniques currently used in cultural heritage science. We use pump-probe microscopy, coupled with a support vector machine, to distinguish common carbon-based black pigments as pure pigments, as two-component black pigment mixtures, and as a mixture of a black and a colorful pigment. This work showcases the potential of pump-probe microscopy to spatially differentiate carbon-based black pigments, which would have interesting applications to works of art.

## Teaser

Pump-probe microscopy, coupled with a support vector machine, identifies carbon-based black pigments in various mixtures.

## INTRODUCTION

There is an unmet need in cultural heritage science for noninvasive identification of carbon-based black pigments, which are broadly used in paintings, drawings, and prints either by themselves or for shading another pigment (*1*). These pigments are produced through controlled burning of a material such as wood, bone, or oil (resulting in charcoal, bone black, and lamp black, respectively) or occur naturally, such as graphite (*2*). They have been identified in some of the oldest pieces of art known to date, such as the cave paintings of Nawarla Gabarnmang in northern Australia (*3*). As their sourcing and cost are not prohibitive, carbon-based black pigments still represent one of the primary black pigment sources.

Material identification is essential for conservation of a work of art and provides insight into its historical context and provenance. In cultural heritage, there are two classification schemes for carbon-based black pigments (*1*, *2*, *4*–*6*). The first is by the form of carbon present in the material, such as graphitic carbons, flame carbons, chars, cokes, or coals. This information is often inaccessible for black pigments incorporated into artwork. The second classification is based on the materials’ origin, such as mineral, vegetable, animal, or soot and smoke, but reliable information for pigments in historic works, particularly carbon-based based pigments where the naming conventions are tangled, is often missing. The two classifications schemes focus on descriptions for carbon-based black pigments and should not be viewed with a lens of modern carbon materials nor modern production methods.

In either classification scheme, carbon-based black pigments are difficult to distinguish by existing methods. The most specific method for identification, scanning electron microscopy with energy-dispersive spectroscopy (SEM-EDS), distinguishes by morphology and, occasionally, elemental composition in pure reference samples (*1*, *2*, *5*, *7*, *8*). However, this requires physical removal of a cross section from the work. Another approach, thermogravimetric analysis and differential scanning calorimetry, can characterize pure reference samples; however, it also requires invasive sampling (*9*). The go-to noninvasive methods in cultural heritage science are linear reflectance techniques, such as fiber-optic reflectance spectroscopy, hyperspectral imaging, multispectral imaging, and Raman spectroscopy due to their ease of use and portability (*10*–*15*). Unfortunately, linear reflectance of carbon-based black pigments is structureless in the visible–near-infrared (NIR) region (*11*, *12*). Raman spectroscopy is a versatile technique that has been shown to be a powerful tool for the characterization of carbons (*16*). The presence of a carbon-based black pigment, in cultural heritage, is confirmed by two characteristic peaks at ~1580 and ~1350 cm^−1^ (*6*, *17*–*20*). The 1580-cm^−1^ peak (G band) is the characteristic Raman peak for crystalline graphite (*6*, *17*). The 1350-cm^−1^ peak (D or disorder band) is used as a measure of disorder in the carbonaceous material; it suggests the presence of heteroatoms in the graphitic structure, in-plane defects, or defects at the edge of the aromatic structure such as a tetrahedral carbon rather than the expected trigonal planar carbon (*6*, *17*). Two studies have delineated pigments using the minute differences between Raman spectra and have applied the findings to cross sections from works of arts (*18*, *20*). Another study used Raman spectroscopy with principal components analysis (PCA) to identify carbonaceous drawing materials (*19*). Raman studies have not yet been expanded to analyze or map mixtures of carbon-based black pigments.

Features in Fourier transform infrared (FTIR) spectra can be used to distinguish between reference carbon-based black pigments (*5*, *21*). However, FTIR spectra from paintings have had mixed results. The spectra are dominated by signals from either the ground layer or the resin varnish; any features that would indicate a carbon-based pigment are overpowered by the other materials present (*22*), or they rely on other compounds present, like hydroxyapatite in ivory and bone black (*23*). X-ray fluorescence (XRF) is another noninvasive technique used in cultural heritage science; it cannot distinguish carbonaceous materials (*24*) but reveals secondary elements like Ca and P in compounds like hydroxyapatite to support identification or rule them out (*25*–*28*). X-ray diffraction (XRD) can differentiate crystalline carbon-based black pigments (like graphite) from noncrystalline forms and can make further distinctions based on noncarbon components similar to XRF (*1*, *2*, *5*, *29*). One group identified hydroxyapatite, indicative of bone black or ivory black, with macroscopic XRD in a still life painting, showing how detection of crystalline, noncarbon components can result in identification (*30*). However, XRD typically cannot differentiate noncrystalline, amorphous carbon-based black pigments (*2*, *4*, *29*). Another study has shown good results using powder XRD and a synchrotron beamline in identifying the type of carbon-based black pigment present in archeological samples but required invasive sampling and powdering of the sample taken (*31*).

Nonlinear optical microscopy techniques, such as two-photon fluorescence, second-harmonic generation, and coherent anti–Stokes Raman microscopy, have been shown to provide noninvasive, high-resolution imaging contrast in applications to biology (*32*) and, more recently, cultural heritage science (*33*–*36*). These contrasts are easily measured because they are emissive, generating light at wavelengths different from the excitation light. However, these conventional multiphoton techniques will not aid in distinguishing carbon-based black pigments; there is little to no fluorescence to analyze (*12*), and nonlinear methods for Raman spectra do not result in different information from spontaneous Raman spectra.

**Fig. 1.**
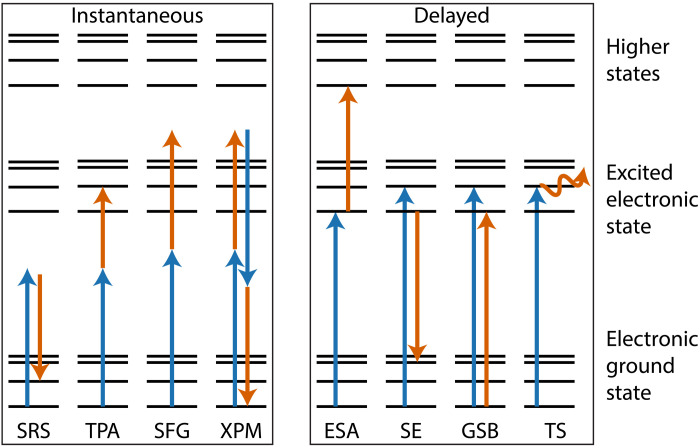
Multiphoton nonlinear processes accessible in P-P microscopy. These interactions modulate the probe laser intensity, generally leading to complex, bipolar TA curves as the time delay is varied. GSB, ground state bleach.

We demonstrate here that another nonlinear optical technique, femtosecond pump-probe (P-P) microscopy, can identify and distinguish common carbon-based black pigments noninvasively. P-P microscopy takes advantage of the nonlinear interactions of two laser pulses with the sample to provide remarkable molecular specificity: In many cases, there are multiple competing molecular mechanisms that provide substantial contrast between nominally similar molecules. We focus here on transient absorption (TA), a subset of P-P, shown in Fig. 1, in which an excitation (pump) pulse affects the absorption of a time-delayed (probe) pulse. “Instantaneous” mechanisms such as stimulated Raman scattering (SRS), two-photon absorption (TPA), sum-frequency generation (SFG), and cross-phase modulation (XPM) give signals only when the pump and probe pulses overlap in time. Other molecular mechanisms result in delayed time signals. The pump laser pulse excites population into higher electronic states, creating a population hole in the electronic ground state. Intermolecular vibrational redistribution rapidly rearranges the population of the electronically excited molecules, which can be transferred by the second pulse into a higher electronic state via excited state absorption (ESA) or to a vibrationally excited level of the ground state through stimulated emission (SE). ESA and SE occur on roughly the same timescale, but the other effects have independent rates. The population hole in the ground state created by the pump pulse reduces the number of molecules available to be excited, reducing the absorption of the probe, a mechanism labeled ground state bleach (GSB). Last, pump absorption can cause localized heating, which, in turn, can change the index of refraction. This affects the scattering profile through the grains of the material, resulting in an effect called thermal scattering (TS). All of these nonemissive P-P interactions are separated from background signals using a modulation transfer scheme, explained in more detail in (*37*). Briefly, the pump laser is amplitude modulated, and a lock-in amplifier is used to detect amplitude modulation transferred to the probe laser; this transfer is only possible if the mechanism involves both pump and probe photons and thus suppresses the, normally larger, linear signal contributions.

Two popular methods have been used in the past to evaluate TA curves and assign them to molecular species: PCA and model fitting (*37*–*43*). Because several nonlinear optical interactions contribute to the measured P-P signals, the resulting TA curves are generally bipolar superpositions of multiple exponential decays and intrinsically nonorthogonal. As PCA works best for linear and orthogonal data structures, we consider it nonideal for identification of carbon-based black pigments. Model fitting of TA curves with exponential basis functions is a powerful method and could allow for pigment identification based on specific lifetimes and amplitudes. However, there exists no method to unambiguously separate the superposition of multiple exponential decays into fundamental components. Also, there are limitations on how precise amplitudes and lifetimes from exponential decays can be extracted for a given signal-to-noise level (*44*, *45*). Because the P-P signals from black pigments are generally weak, spatial resolution would need to be sacrificed by downsampling to achieve an appropriate signal-to-noise level for fitting. Therefore, we opt for alternative methods better suited to handle the weak signals typical of black pigments.

P-P microscopy has successfully been applied in a wide range of applications, including melanin characterization in biological tissue (*38*). This application provides a good example of its versatility: The melanin absorption spectrum is broad and featureless, but P-P images reveal notable heterogeneity from many competing molecular mechanisms shown in Fig. 1, and the contrast correlates with disease progression in melanoma. Previous cultural heritage applications include identification of iron oxides and red organic dyes, visualization of vermilion and cadmium yellow degradation, and as a tool to noninvasively obtain a virtual cross section of historical works of art (*46*–*51*). Here, we use P-P microscopy to identify and analyze four of the most used carbon-based black pigments: bone black, charcoal, graphite, and lamp black. We demonstrate that P-P microscopy resolves nonlinear features of these pigments that allow identification in two-compound black-black mixtures, applicable in separating an underdrawing from black paint used in upper layers and to identify black pigments in shading applications, i.e., a mixture of black with different colored pigments, such as ultramarine blue.

For cultural heritage applications, a specific region or volume of interest is imaged to derive pigment identity maps or abundance maps. This is similar to methods used in hyperspectral or Raman imaging, where unmixing algorithms determine the proportion of an a priori known reference spectrum within every pixel of the sample’s image. In the remainder of this manuscript, we present how we acquired and evaluated P-P image stacks of pure pigment paints and paints that are mixtures of two different pigments. First, we use P-P image stacks of pure pigments (reference samples) to train a classifier algorithm. This classifier algorithm is then used to classify P-P image stacks of two-pigment mixtures. We use an unmixing algorithm as a baseline and compare it to a support vector machine (SVM) that shows considerably better performance in identifying pigments. In a final section, we discuss our results, their limitations, and provide an outlook into next steps.

## RESULTS

### P-P spectroscopic features of black pigments

**Fig. 2.**
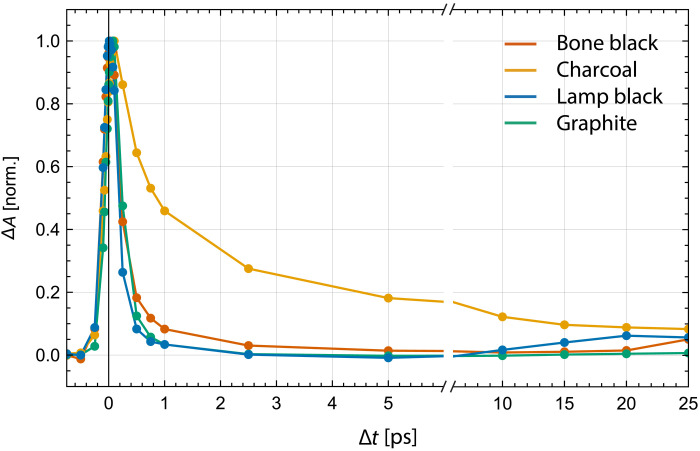
Spatially averaged P-P TA curves of bone black, charcoal, graphite, and lamp black.

We acquired reference P-P image stacks of pure paint samples of bone black, charcoal, graphite, lamp black, and ultramarine blue with a pump wavelength of λ_pump_ = 720 nm and a probe wavelength of λ_probe_ = 817 nm. The pigments were validated with reflectance spectroscopy, elemental analysis, and Raman spectroscopy (see figs. S1 to S7 and table S1). P-P data from the image stacks of pure pigments were averaged across the spatial dimensions, which are over the imaged field of view, and normalized. These curves are shown in Fig. 2, and the curve for ultramarine blue is shown in fig. S8. The nonnormalized TA curves can be found in the Supplementary Materials (fig. S9). The TA curves of the four black pigments exhibit distinctive qualitative differences. For graphite and lamp black, the duration of the temporal features (≈100 fs) is limited by the temporal resolution of our microscope. These instantaneous signals suggest the involvement of virtual energy states in the nonlinear interaction, typical of processes like TPA, SRS, SFG, and XPM, as shown on the left in Fig. 1. In our convention, transient loss processes, such as TPA, are depicted as positive while transient gain processes are depicted as negative. For the given pump and probe wavelength, we would only detect Raman gain processes, thereby ruling SRS out as the potential signal origin. SFG generates a different wavelength, which we have not observed, and XPM manifests in a sign change in the TA signal. This indicates TPA as the likely signal origin for graphite, bone black, and lamp black around the time delay of Δ*t* = 0 ps. In the case of lamp black, there is a slow rise in the TA curve observable at time delays larger than 15 ps. This is caused by an ESA event with a lifetime much longer than the observed 25 ps. In addition to TPA, bone black contains an ESA process with a short lifetime, visible between 1 and 5 ps. Charcoal signals are dominated by multiple ESA processes, which are described by a superposition of multiple exponential decays. The differences in the TA curves highlight the potential of P-P microscopy to noninvasively identify and distinguish these four carbon-based black pigments. It is worth noting that these four curves, particularly graphite, also differ in amplitude as shown in fig. S9.

**Fig. 3.**
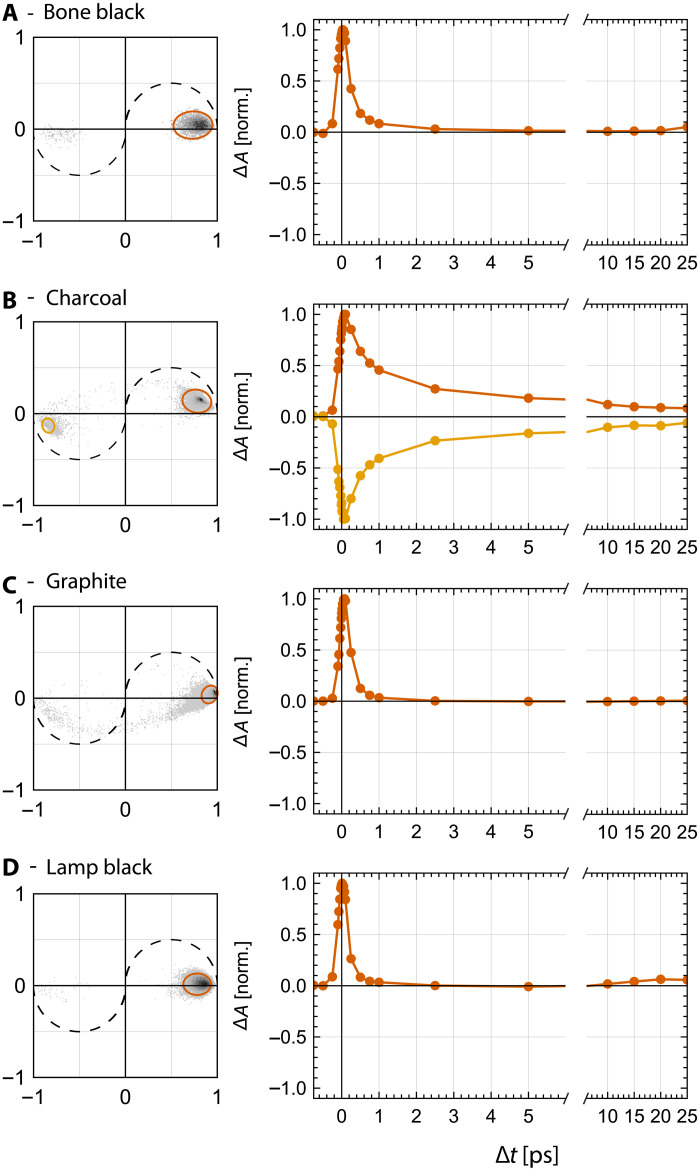
Phasor plots and P-P signal components of the carbon-based black pigments. (**A** to **D**) Left: The phasor coordinates of all signal-containing pixels in pure pigments (bone black, charcoal, graphite, and lamp black, respectively) as histograms, computed with a phasor frequency of *f* = 0.25 THz. Right: Averaged P-P signals corresponding to the circled regions of the phasor plot.

The spatially averaged curves shown in Fig. 2 obscure signal variations within the P-P image. For example, the averaged TA curve of charcoal is uniformly positive, peaking around a time delay of Δ*t* = 0.1 ps. However, high-resolution P-P images, shown in fig. S10, reveal interspersed regions of positive and negative signal for charcoal. A convenient way of visualizing heterogeneity in P-P stacks is an adapted form of phasor analysis (*52*) (see Materials and Methods). Nearby points in a phasor plot correspond to similar P-P signals. Phasor plots of the pure black pigments are shown in Fig. 3. It is evident that the phasor plot for charcoal falls into two distinct areas, aligning with positive and negative P-P curves, respectively. We select all TA curves within these clusters, indicated by red and yellow circles, and plot their averages in Fig. 3B, respectively. The signals in charcoal appear to be the same aside from a sign difference. This suggests TS as an underlying molecular mechanism: A pump-induced change in refractive index transiently changes the angular distribution of the backscattered light, which, in combination with an aperture in the beam path, causes a sign change in the measured signal. An alternative interpretation would be the presence of two distinct chemical species in charcoal, which coincidentally have nearly opposite signs at each delay. While bone black would be the pigment expected to show signals for two distinct chemical species, as it contains both the carbonized organic material of the bone and the inorganic hydroxyapatite (*1*, *2*), we do not see this present in the P-P images. Conversely, P-P signals of bone black, graphite, and lamp black appear homogeneous in their phasor plots.

We also acquired P-P image stacks of paints that are composed of two different pigments. These include six 50-50 (by mass) pigment mixtures of each pair of two black pigments: bone black–charcoal, bone black–graphite, bone black–lamp black, charcoal-graphite, charcoal-lamp black, and graphite–lamp black. Bright-field images can be found in fig. S11. We acquired multiple P-P images from different areas of each mixture. Their average TA curves are shown in fig. S12. To demonstrate pigment identification in shading applications, we imaged mixtures of each carbon-based black pigment with synthetic ultramarine blue (Na_7_Al_6_Si_6_O_24_S_3_), an artificial version of natural ultramarine blue that comes from the mineral lapis lazuli. We captured images of 12 combinations, mixing each of the four black pigments with ultramarine blue in three paint ratios: 25:75, 50:50, and 75:25. Bright-field images of all mixtures can be found in fig. S13, and average TA curves are shown in fig. S14. On the basis of the reference P-P image stacks, we characterize the pigments in the mixtures using an unmixing algorithm and an SVM to identify the pigments composing the mixtures.

### Pigment assignment by unmixing

The averaged TA curves of the pigments, shown in Fig. 2, show distinct features that we will use to distinguish them in paint samples that are made of two pigments. We use the averaged P-P curves from pure pigments shown in Fig. 3 as reference TA curves for the unmixing algorithm. This includes two reference curves for charcoal, due to its noted heterogeneity. Phasor analysis is used to derive the two charcoal reference curves, the orange and yellow TA curves shown in Fig. 3B. The unmixing algorithm analyzes the TA curves of all pixels in a P-P image stack. It compares these curves with the reference curves from pure pigments and determines the abundance fraction, which represents the proportion of each reference pigment present in every pixel. Subsequently, after the unmixing process, abundance fractions for the two reference TA curves associated with charcoal are combined to a single charcoal abundance. More details can be found in Materials and Methods.

The unmixing algorithm is first tested on pure pigment data and has an average accuracy of 83%. This means that, when the unmixing algorithm is presented with a P-P TA curve from a single pixel of any of the five pigments (bone black, lamp black, charcoal, graphite, and ultramarine blue), it classifies it correctly 83 times of 100. Because of the similarity of bone black and lamp black signals, we repeated the unmixing analysis with a reduced set of pigments. First, we only considered lamp black, charcoal, graphite, and ultramarine blue (unmix-nobb), and in a second run, we only considered bone black, charcoal, graphite, and ultramarine blue (unmix-nolb). The unmixing classification accuracy for pure pigments improves to 88% (92%) when bone black (lamp black) is left out.

When presented with P-P image stacks of black pigment mixtures, the unmixing approach identified only 63% of pixels as being pigments that were actually present in the sample for the black-black mixtures and only 62% in the ultramarine blue–black mixtures. As above, we repeated the unmixing analysis with a reduced set of pigments in the unmixing algorithm (unmix-nobb and unmix-nolb). When bone black is left out, the unmixing approach correctly identified only 76% of the pixels in black-black mixtures and 72% in the ultramarine blue–black mixtures. When lamp black is left out, the unmixing approach correctly identified only 69% of the pixels in black-black mixtures and 64% in the ultramarine blue–black mixtures. The signal-to-noise ratio (SNR) in P-P data of black pigments is relatively low, increasing the variance in single-pixel data. We believe this to be a main limitation as spectral unmixing compares these noisy single-pixel spectra to highly averaged reference spectra (see fig. S15), which may not handle the noise effectively. Given the low percentage of correctly identified pixels, we changed our approach to methods that are better suited to the complex and heterogeneous nature of the black pigment P-P data. We summarized accuracies of all algorithms across this manuscript in table S2 in the Supplementary Materials.

### Machine learning for pigment classification

Because of the poor performance of the unmixing algorithm, the high dimensionality of TA curves, and the signal heterogeneity of charcoal, we decided to go a different route and train an SVM for classification. We trained an SVM with P-P TA curves from pure pigments and then used it to classify and identify pigments in two-component mixtures. We train the SVM with around 5500 TA curves of each of the five pigments exposing the SVM to the full range of signal variation for each pigment. A description of the training, validation, and testing process can be found in Materials and Methods.

The resulting SVM has an overall accuracy of 85% for pure pigments. Because of the similarity of bone black and lamp black signals and analogous to the unmixing case, we repeat the same procedure with reduced pigment sets: First, bone black is left out, and in a second run, lamp black is left out. The accuracy increases markedly in both cases to 96% (no bone black) and to 94% (no lamp black). However, the performance of the SVM will drop for P-P images of two-pigment mixtures, as discussed in the next sections. In the remainder of this manuscript, unless explicitly stated otherwise, “SVM” refers to the classifier trained on all pigments. If we refer to a classifier trained by leaving one pigment out, we will specify it as “SVM-nobb” (no bone black) or “SVM-nolb” (no lamp black). We summarized accuracies of all algorithms across this manuscript in table S2 in the Supplementary Materials.

### Black-black mixtures

The accuracies reported above were based on the pure reference samples, and we want to test the SVM on a more realistic scenario, namely, paints based on two-pigment mixtures: bone black–charcoal, bone black–graphite, bone black–lamp black, charcoal-graphite, charcoal–lamp black, and graphite–lamp black. We took images of at least three different areas for each mixture and presented the P-P image stacks to the SVM classifier.

**Fig. 4.**
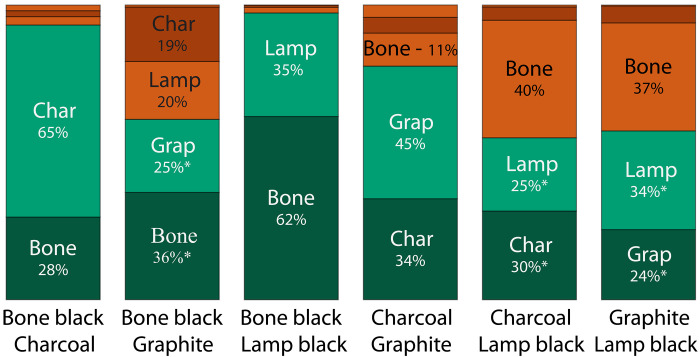
Summary of SVM performance on two-black pigment mixtures. The bar charts display the breakdown for classification of each black-black mixture. The full bar represents 100%. Green corresponds to correctly classified pixels. Red corresponds to misclassified pixels. Percentages with an asterisk denote mixtures that have misclassification at comparable percentages of correct classifications. These mixtures perform better with the appropriate reduced pigment classifier.

We summarize the overall performance of the SVM classifier with a bar chart in Fig. 4, one bar for each black-black mixture. It is crucial to note that we operate without a definitive “ground truth” in this context. While we know the two pigments comprising each mixture, their precise microscopic distribution remains unknown, and no alternative method exists for validating the results, to the best of our knowledge. To assess performance, we tally all classified pigments for a given mixture. We label pixels as correctly classified if they are identified as a pigment that is part of the mixture and as misclassified if not. Accuracy is computed by taking the ratio of correctly classified pixels over all classified pixels. A successful method to identify black pigments should have a high accuracy, meaning that the percentages of correctly identified pixels should be larger than any other misclassification. The SVM achieves accuracies of 55% (charcoal–lamp black), 57% (graphite–lamp black), 61% (bone black–graphite), 79% (charcoal-graphite), 93% (bone black–charcoal), and 97% (bone black–lamp black). The first three accuracies are low and stem from the similarity of bone black and lamp black signals. By pivoting to SVM-nobb and SVM-nolb, these accuracies are lifted to 94% (charcoal–lamp black with SVM-nobb), 89% (graphite–lamp black with SVM-nobb), and 79% (bone black–graphite with SVM-nolb). A detailed list is presented in the Supplementary Materials (table S3). We will discuss the implications of these results in Discussion.

**Fig. 5.**
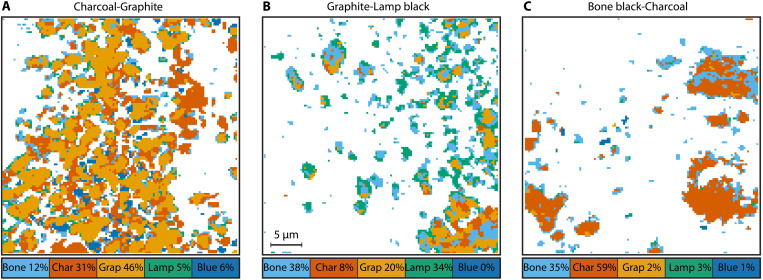
Pigment map for three black-black mixtures. (**A**) Charcoal-graphite. (**B**) Graphite–lamp black. (**C**) Bone black–charcoal. The percentages are for these specific fields of view and deviate from the average for each mixture.

The SVM returns a pigment classification for each pixel in the P-P stack, and we use this information to generate a false-color pigment map (or abundance map). Three representative examples are shown in Fig. 5. The percentages in the legend represent pigments identified by the classifier. Note that these numbers represent one specific region of interest and therefore deviate from the overall average. For the charcoal-graphite mixture in Fig. 5A, 77% of the signal containing pixels are identified as charcoal (31%) or graphite (46%). The remaining 23% were misclassified, with 12% of the pixels misclassified as bone black. The graphite–lamp black mixture, shown in Fig. 5B, performs poorly with only 54% of pixels being correctly identified (20% graphite and 34% lamp black). The largest misclassification is again bone black (38%). This makes sense because of the similar signals for lamp black and bone black. If, instead, SVM-nobb is used to classify the same image stack, then we find 85% of pixels correctly identified. The direct comparison between abundance maps of SVM and SVM-nobb for this region of interest is shown in fig. S16. In the bone black–charcoal mixture, Fig. 5C, 94% of pixels are accurately identified (35% bone black and 59% charcoal) and with the most common misclassification being lamp black, with only 3% of the pixels.

### Ultramarine blue-black mixtures

**Fig. 6.**
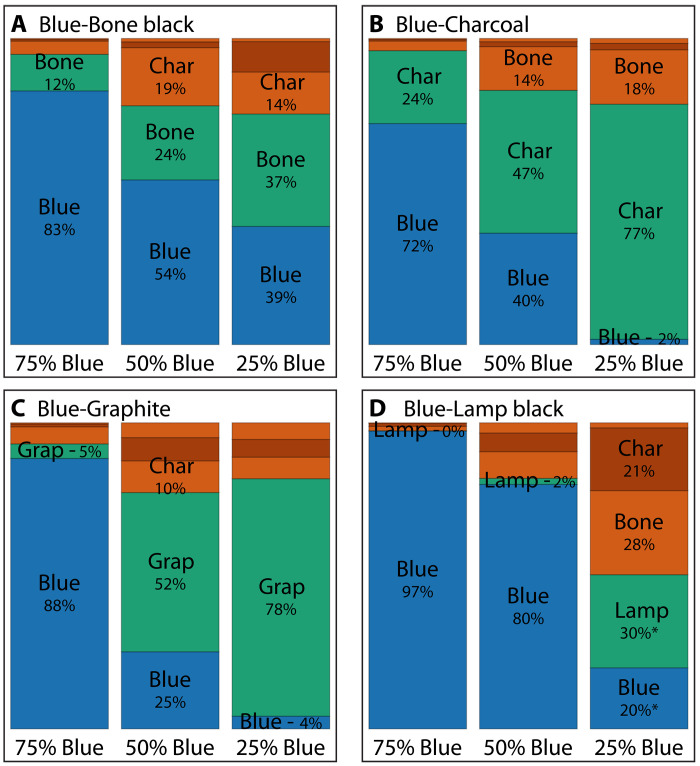
Summary of SVM performance on blue-black mixtures. (**A**) Bone black–ultramarine blue. (**B**) Charcoal–ultramarine blue. (**C**) Graphite–ultramarine blue. (**D**) Lamp black–ultramarine blue. The full bar represents 100%. Blue and green correspond to correctly classified pixels, blue for ultramarine blue and green for the black pigment. Red corresponds to the misclassified pixels.

We also tested the SVM classification performance for shading applications on mixtures of a carbon-based black pigment with ultramarine blue. We presented 12 mixtures of a carbon-based black pigment and ultramarine blue in three different paint ratios to the SVM classifier. For each of the 12 samples, we imaged at least three different areas and computed the average classification accuracy. A summary of the classifier across all the shading combinations is presented in Fig. 6 (see table S4 for full details). Like in the black-black mixture case, there exists no ground truth for the pixel identities, and we therefore apply the same metric to measure accuracy. Our methodology yields a robust qualitative classification, demonstrating a strong correlation between the detected amount of ultramarine blue and the actual physical mixing ratio of the sample. This correlation is consistent for most of the black pigments used. Of the 12 mixtures, only the 25:75 ultramarine blue–lamp black mixture performs poorly with only 50% of all pixels correctly classified. Four mixtures (50:50 ultramarine blue–bone black, 25:75 ultramarine blue–bone black, 25:75 ultramarine blue–charcoal, and 50:50 ultramarine blue–graphite) are classified with an accuracy ranging between 75 and 80%, and all remaining mixtures are classified with a higher than 80% accuracy. As before, the alternative classifier SVM-nobb improves the classification accuracy for the 25:75 ultramarine blue–lamp black mixture to 64%.

**Fig. 7.**
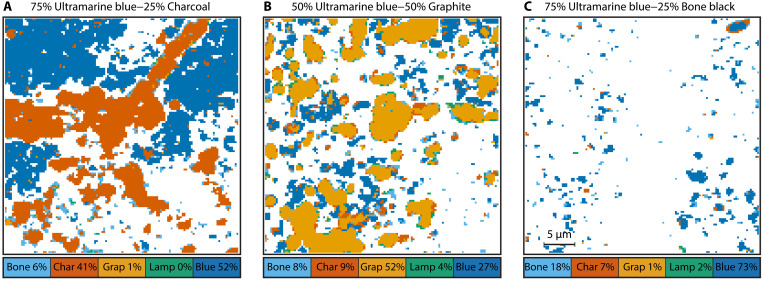
Pigment map for three ultramarine blue–black mixtures. (**A**) 75:25 ultramarine blue–charcoal. (**B**) 50:50 ultramarine blue–graphite. (**C**) 50:50 ultramarine blue–bone black. The percentages are for these specific fields of view and deviate from the average for each mixture.

Like with the black-black mixtures, we can derive spatial maps of the black-blue mixtures. Three maps, of the 75:25 ultramarine blue–charcoal, 50:50 ultramarine blue–graphite, and 50:50 ultramarine blue–bone black mixtures, are shown in Fig. 7. As before, the derived mixing ratios deviate from the macroscopic mixing ratio, highlighting that, without a true ground truth, genuine quantitative imaging remains a challenge. The images shown in Fig. 7, and the bar chart in Fig. 6, demonstrate good qualitative performance.

## DISCUSSION

Our findings highlight the potential of P-P microscopy for the noninvasive differentiation and mapping of black pigments. This technology presents an appreciable advancement in the noninvasive analysis of cultural heritage artifacts, where the precise identification of pigments can provide invaluable insights into the techniques and materials used by artists. However, there are cases of misclassification within the set of targets. Here, we will discuss the performance and limitations of our current classification approach and comment on the future application of P-P microscopy in cultural heritage science.

### Classification challenges and future strategies

Our current classification approach faces several challenges. The primary challenge we encounter with our current classification approach is the absence of a definitive ground truth for the images. The derived pigments maps, as shown in Fig. 5, are a result of P-P imaging and an SVM classifier. To the best knowledge of the authors, there is no alternative method to validate the correct mapping for all pigments. The only ground truth we have is the mixing ratio used during sample preparation. The mixing ratio is a macroscopic quantity, and the P-P images in this proof-of-principle study only sample three areas of 36 μm × 36 μm. Within this small area, we expect variations in the pigment ratios that will vary from the macroscopic pigment distribution. For example, the measured pigment ratio in the 50:50 bone black–charcoal mixture, in Fig. 5, is 35/59 ≈ 0.6, which could be explained by a locally higher density of charcoal.

A second challenge in pigment classification is the vast difference in SNR between different pigments. We show unnormalized curves of the four black pigments in the Supplementary Materials (fig. S9). Graphite has the largest signal followed by ultramarine blue and charcoal, which are around five times weaker. Lamp black is roughly 10 times weaker and bone black around 20 times weaker than graphite. Signals of bone black and lamp black are close to the noise floor of our microscope and are therefore more difficult to classify compared to the high SNR signals of charcoal and graphite. We believe that this is the main reason for the small bone black percentages identified in black-black mixtures, depicted in Fig. 4. We hypothesize that most misclassified pixels stem from the SNR limitations. We have plotted five randomly selected single-pixel TA curves per reference pigment and overlaid them with their respective average curve in fig. S15. Some of these TA curves contain sizable noise and qualitatively differ from their respective average curves. This also relates to the inherent trade-off between the spatial resolution and SNR: Higher spatial resolution requires smaller sampling volumes and therefore leads to a smaller SNR. Averaging neighboring pixels in an image increases the SNR at the expense of spatial resolution. Spatial averaging, however, might mix TA curves of adjacent pigments. This causes two additional challenges. In the case of a large signal amplitude mismatch, the pigment with the larger signal will overwhelm the features of the weaker pigment signal, thereby skewing classification toward pigments with the larger signal. Thus, even for a 50:50 mixture of two different pigments, their difference in signal strength can distort the pigment distribution that we measure toward the pigment with the larger signal. This effect can be seen in the mixtures of ultramarine blue with either bone black or lamp black, where the percentage of classified ultramarine is typically larger than the mixing ratio. Alternatively, if TA curves of comparable signal strengths are averaged, then we create a de facto new TA curve that is unknown to the SVM classifier, which was only trained on pure TA curves, leading to increased misclassification. Now, we choose a compromise between spatial averaging to achieve sufficient SNR and maintaining enough resolution to resolve most individual pigment grains.

Another reason for misclassification of TA curves is the inherent similarity between the signals of bone black and lamp black. The highly averaged curves of their pure reference samples, as shown in Fig. 2, only differ marginally in decay time between 1 and 5 ps and in their offset at time delays larger than 15 ps. The small signal amplitudes of lamp and bone black are not prominent enough on a pixel-by-pixel basis and cause misclassifications in the SVM classifier. This effect can be seen most prominently in the 50:50 charcoal–lamp black mixture where 40% of all pixels are misclassified as bone black and in the 50:50 graphite–lamp black mixture where 37% of all pixels are misclassified as bone black. To mitigate this problem, we introduced the additional classifiers SVM-nobb and SVM-nolb by leaving out bone black and lamp black, respectively. These classifiers perform notably better in mixtures of two black pigments; however, additional a priori knowledge of the sample would be required to select the appropriate classifier. This is only a limitation when restricted to solely use P-P microscopy. The presence of calcium and phosphorus in bone black offers another route to unambiguously distinguish bone black from lamp black. XRF spectroscopy can noninvasively detect both elements and could be used to decide if the SVM-nobb or SVM-nolb classifier should be used for further analysis.

In the future, we envision increasing the SNR in two ways: First, by improving our detection capabilities, specifically by increasing collection efficiency by using higher–numerical aperture (NA) objectives. Second, we intend to explore different pump and probe wavelengths that might not only offer different TA dynamics that could be used to distinguish bone black from lamp black but also offer a larger interaction cross section for the currently weak signals of bone black and lamp black.

Our current classification scheme uses a relatively simple algorithm that analyzes individual pixels independently, without considering the contextual information from neighboring pixels. While SVMs provide robust performance in classifying TA curves on a per-pixel basis, the inherent spatial correlation within pigment grains suggests that adjacent pixels are likely to belong to the same pigment. This spatial dependency is not used in the current approach, potentially limiting the overall classification accuracy. Given the high probability that adjacent pixels represent the same pigment, leveraging this local image information could substantially enhance classification performance. Convolutional neural networks (CNNs) are particularly well suited for this task as they are designed to capture spatial hierarchies in data through convolutional layers that process local neighborhoods of pixels. Thus, we envision implementing U-Net, a specialized CNN for semantic segmentation to integrate spatial context into the classification process to further improve classification accuracy and robustness of pigment identification (*53*).

We also observe, at least heuristically, an increased misclassification rate at pigment grain boundaries. This can be seen in Figs. 5A and 7A, in which bone black appears around the edges of individual pigment grains. This can also be observed for other pigments such as lamp black. We attribute this to the qualitatively similar curves of bone black, lamp black, and graphite. TA curves originating from grain boundaries are weaker because there is less material in the focal volume of the lasers to contribute to P-P signals. This could cause a graphite signal to be misclassified as either bone black or lamp black, for example. We are confident that this problem can be circumvented by algorithms that take information from the pixel neighborhood into account, like CNNs.

### P-P signal heterogeneity

An unexpected discovery was the intrinsic heterogeneity in the P-P signals of charcoal. As shown with phasor in Fig. 3B, charcoal has two distinct P-P signals: a positive and a negative signal. In addition to being the only pigment with a distinctive lifetime present in its positive signal longer than 5 ps, the presence of a negative signal is also unique among the carbon-based black pigments studied. We hypothesize that two factors contribute to potential heterogeneity: First, the inherent microscopic heterogeneity of charcoal. Of the four pigments, only graphite has an ordered molecular structure (sheets of sp^2^-hybridized carbon), consistent with the observed homogeneous signal in the phasor plot in Fig. 3C. Lamp black, which undergoes a gas phase carbonization during production, is microscopically uniform (on the scale of the resolution of our microscope). Charcoal is derived from an extremely heterogeneous base material, wood, maintains a solid structure during carbonization, and therefore retains part of its initial structural complexity. While bone black also derives from a heterogeneous starting material, bone, it undergoes a liquid phase during carbonization, which would allow for some molecular rearrangement, with the resulting pigment being more homogeneous. The other factor that could contribute to the heterogeneity in P-P signals of charcoal is the presence of heteroatoms or noncarbon constituents that commonly occur in carbon-based black pigments. Winter reports that incorporation of heteroatoms into the carbon matrix during the carbonization process is especially common for cokes and chars prepared at low temperatures (*2*). These factors may explain the higher degree of heterogeneity of signals in charcoal. Again, bone black notably has heteroatoms present in the form of hydroxyapatite, but we do not observe heterogeneity in its signal. The potential of P-P microscopy to analyze the heterogeneity of carbon-based black pigments is an exciting prospect for future studies.

### Beyond proof-of-principle studies toward applications to works of art

This manuscript demonstrates the potential of P-P microscopy to noninvasively identify black pigments in mixtures. For this proof-of-principle demonstration, we restricted ourselves to four black pigments and one colored pigment. Most works of art contain many more colors, and although we used the four most prevalent black pigments, there are other black pigments in use. Our group has analyzed a range of pigments, including red organic dyes, iron oxides, vermillion, and cadmium sulfide, and we can incorporate these pigments into our classification scheme (*46*–*51*). Furthermore, P-P microscopy offers two powerful and easily accessible degrees of freedom: the choice of pump and probe wavelength. P-P signals reflect the population dynamics between molecular levels and are therefore strongly dependent on the pump and probe wavelengths. Pigments that present similar TA curves at a particular wavelength combination may differ drastically at another (*47*). A convenient approach would be pigment exploration in a broadband P-P spectroscopy setup, where many wavelengths can be probed simultaneously. We could then select a wavelength combination that offers a unique contrast for a specific pigment. Ultimately, multiple P-P images acquired with different wavelength combinations (hyperspectral P-P microscopy) will provide sufficient specificity to distinguish and identify many pigments. Extension of the SVM classifier to more pigments and to hyperspectral P-P images is conceptually straightforward and only requires the additional pure reference data in the training phase. In addition, polarization P-P microscopy, which offers improved chemically specific contrast based on the molecular anisotropy of pigments, can further improve pigment specificity (*38*, *54*).

The multiphoton nature of P-P microscopy enables high resolution in all three spatial dimensions, even beneath the surface of highly scattering materials (*32*). In previous experiments, we were able to image up to a depth of ≈90 μm in paint layers to produce virtual cross sections (*49*), thus allowing cultural heritage scientists and conservators to better understand pieces of art without invasive sampling. However, achievable penetration depths depend on the absorption and scattering properties of the materials present at the surface and the subsequent layers. Carbon-based black pigments strongly absorb visible-to-NIR light and therefore reduce optical penetration depth. This will be most prominent in works of art with a thick or opaque layer of carbon-based black paint, for example, in oil or tempera paintings. In a work with thinner or more transparent layers, such as watercolor paintings or drawings and prints, the absorption of the black pigments would not greatly reduce penetration depth.

Our study has successfully shown that P-P microscopy is an effective noninvasive tool for differentiating black pigments in a variety of combinations, including mixtures with other carbon-based black pigments and with ultramarine blue. This achievement highlights P-P microscopy’s capability to fill a void in the field of cultural heritage science, where, until now, no noninvasive method for identifying carbon-based black pigments in mixtures with such certainty existed. We have outlined a clear strategy to further improve the performance and to increase the number of pigments in our approach and we envision applying this methodology to actual works of art. A particularly fascinating application would be Vermeer’s *Girl with a Pearl Earring* where bone black and charcoal are reported to exist together in an underlayer, currently only confirmed by analysis of a cross section (*55*). P-P microscopy could be used to further validate these findings as well as to provide additional information, i.e., a three-dimensional pigment map of both pigments across the painting.

## MATERIALS AND METHODS

### P-P microscopy

A schematic of our P-P microscope is shown in fig. S17. The output of a Ti:sapphire laser (Coherent Chameleon Ultra II) with an 80-MHz repetition rate is split into two parts. One part serves as a probe beam at a wavelength of λ_probe_ = 817 nm. The second part is frequency converted into the pump with a wavelength of λ_pump_ = 720 nm with an optical parametric oscillator (Coherent Mira-OPO). The pump pulse train is intensity modulated by an acousto-optic modulator at a rate of 2 MHz. Both laser beams are spatially superimposed, sent into a laser scanning microscope, and focused onto the sample with a 20x 0.7-NA dry objective. The interpulse delay Δ*t* between the pump and probe is controlled with a motorized translation stage in the probe beam path. We use a modulation transfer scheme to detect the weak signals generated by the nonlinear interaction between the pump, probe, and sample. As the nonlinear interaction transfers the pump modulation onto the probe pulse train, these changes in absorption in the probe pulse train are measured with a photodiode and a lock-in amplifier. For pigment imaging, we use a pump and probe pulse intensity of *I* = 4.4 × 10^8^ W/m^2^, (corresponding to 0.25 mW) and image an area of 36 μm × 36 μm for 24 time delays Δ*t* spanning −1.5 to 25 ps. The resulting data structure (image stack) is a three-dimensional data cube with two spatial and one temporal dimension. Each pixel in the P-P stack represents a P-P TA curve, the change in absorption as a function interpulse delay Δ*t*.

### Validation of pigments

For reflectance spectroscopy, pigment was placed onto a glass slide and fixed with a gum arabic solution. A coverslip was placed over the sample and allowed to dry in the fume hood overnight. Reflectance spectra were collected using a Cary 5000 spectrophotometer with a diffuse reflectance accessory. The reflectance measurements include both specular and diffuse reflectance. The spectra were collected from 400 to 1500 nm with a step size of 1 nm and a scan time of 0.1 s. A Labsphere diffuse reflectance standard was used as a reference.

For elemental analysis, double-sided copper tape was placed on a sample mount for the instrument. Pigment powder was pressed onto this tape, with excess removed via nitrogen gas flow. The pigments remained uncoated. An Apreo S SEM by Thermo Fisher Scientific with an Oxford Instruments X-Max-N 150 EDS was used for elemental analysis with an accelerating voltage of 20 kV. Because of the copper tape, copper does appear in the elemental spectra for the pigments analyzed.

For Raman spectroscopy, the pigment was placed onto a glass slide and held in place by a glass coverslip. Raman spectra were collected using a Horiba Jobin Yvon LabRAM ARAMIS Raman microscope with an air-cooled (−70°C) charge-coupled device detector. A grating of 1800 g/mm and a slit of 100 μm were used with a 50x objective and a wavelength of 633 nm. The spectral resolution was ~1 cm^−1^. The spectra were collected from 1050 to 1800 cm^−1^. Each spectrum was averaged 35 times with each acquisition being 30 s, resulting in a total scan time of 18 min.

### Preparation of pigments

The pigments were commercially sourced from AGS Company (graphite), Coates Charcoal (charcoal), Kremer Pigments (bone black, exclusive and ultramarine blue, and dark), and Rublev Colours (lamp black). Pure pigments were thoroughly mixed with gum arabic in a separate vessel to prepare a smooth watercolor paint. For the black-black mixtures, the powdered pigments were weighed and mixed with a mortar and pestle as powders and then together mixed with gum arabic to prepare the watercolor paint. The densities of the pigments were measured by packing the individual pigment into a known volume, 0.5 ml, and measuring the weight. The measured densities are as follows: bone black, 0.71 g/cm^3^; charcoal, 0.36 g/cm^3^; graphite, 0.74 g/cm^3^; and lamp black, 0.33 g/cm^3^. For ultramarine blue–black mixtures, the paints were prepared separately as described for pure pigments and then mixed together as one would on a palette. The prepared paints were applied to a commercially sourced preprimed canvas in two layers, allowing for drying in between. Note that gum arabic itself does not cause P-P signals, as shown in fig. S18 in the Supplementary Materials.

### Adapted phasor analysis for visualization

A more detailed description of adapted phasor analysis can be found in (*52*). In brief, single-frequency sine and cosine Fourier coefficients are calculated for TA curves in each pixel of a P-P image stack and plotted as the *x* and *y* coordinates on a two-dimensional plane, the phasor plot. For example, phasor coordinates of a positive (negative) single-exponential decay would map onto a specific point on the semicircle in the first quadrant (third quadrant). Nearby points in a phasor diagram correspond to similar P-P signals. Thus, adapted phasor analysis provides a simple way of visualizing the inherently three-dimensional P-P image stacks. The phasor frequency, the frequency for which the Fourier components are calculated, is a degree of freedom that can be adjusted to tune the phasor plot, i.e., to be more sensitive to specific timescales in the TA data. For the black pigment data, we use a frequency of *f* = 0.25 THz, which nicely separates the signals of charcoal into two distinct areas in the phasor plot. The first area is in the first quadrant and corresponds to the positive charcoal signals, while the second area falls into the third quadrant, which corresponds to the negative charcoal signals. Adapted phasor analysis thus provides a convenient way of separating these signals based on their position in the phasor plot. The positive (negative) TA curve shown in red (yellow) in Fig. 3B corresponds to all pixels that are selected with the red (yellow) ellipse in the phasor plot.

### Data analysis and classification algorithms

The goal of our data analysis is to find a classifier function that predicts pigments based on TA curves. After data preprocessing, we use P-P image stacks of pure pigments (reference samples) to train a classifier algorithm. This classifier algorithm is then used to classify P-P image stacks of two-pigment mixtures. In many similar scenarios such as hyperspectral imaging or Raman imaging, where the spectra of reference samples are well known, unmixing algorithms are the standard approach. Thus, we decided to use an unmixing algorithm as a baseline and compare it with an SVM, which is more suited to deal with heterogeneous data such as TA curves. Here, we describe the data preprocessing and training in more detail.

#### Data preprocessing of P-P image stacks

Raw P-P data are preprocessed before training and classification in the following steps: (i) Because of pump leakage into the detector and potential long-lived (τ >> 12.5 ns, the time spacing between consecutive pulses) radiative states at the probe wavelength, we average three P-P images at negative time delays (Δ*t* = −10, −5, and −2.5 ps) and subtract them from the entire P-P stack, thereby eliminating a constant offset in the data. These three time delays are then removed from the image, resulting in the 24 time delays mentioned in the main text, to reduce the dimensionality for machine learning, improving training and classification speed. (ii) Raw P-P data are intentionally oversampled beyond the diffraction limit, and we apply a spatial moving average filter of kernel size two to increase the SNR. (iii) A global intensity threshold is applied to all P-P stacks to discriminate noise from P-P signals. The threshold is based on the maximum in the histogram of all P-P stack projections. (iv) We then reduce the image size with an average pooling by a factor of 2, consistent with the oversampling, to reduce data amount and increase training and classification speed.

#### Unmixing algorithm

TA curves of pigments ultramarine blue, bone black, graphite, and lamp black are homogeneous, and their reference P-P stacks are spatially averaged to reference TA curves, as shown in Fig. 3 and fig. S8. Charcoal is the only pigment showing appreciable signal heterogeneity, containing both negative and positive TA curves (see Fig. 3B). We use adapted phasor analysis to derive two reference TA curves for charcoal, as described in the “Adapted phasor analysis for visualization” section of Materials and Methods. All TA curves, averaged reference curves and single-pixel curves of mixtures, are normalized to their respective extremum. The reference curves for each pigment are arranged into an endmember matrix *U*. The unmixing algorithm uses the endmember signatures in matrix *U* to perform a fully constrained least-squares fit on each pixel, determining the proportion of each reference pigment in every pixel. “Fully constrained” incorporates a nonnegativity constraint, which permits only positive values in the abundance fractions and a “sum-to-1” constraint requiring the sum over the abundance fractions to be 1. This allows interpretation of the abundance fraction as probability, and we assign the pigment with the highest probability to a given pixel. The algorithm used in this manuscript is based on (*56*) and was implemented in pysptools 0.15.0 by C. Therien (*57*).

To assess the performance of the unmixing algorithm, we randomly split the pure pigment data in a 50:50 ratio into the test and train set. The train set is used to compute average reference TA curves, and these curves are then used to unmix the test data. The test accuracy *acc*_test_ describes the fraction of correctly identified TA curves in the test data. We repeat this procedure five times to compute the mean value and SD of the test accuracy. We use three unmixing algorithms: unmix, which contains reference TA curves of all five pigments (bone black, charcoal, graphite, lamp black, and ultramarine blue); unmix-nobb, without the pigment bone black; and unmix-nolb, without the pigment lamp black. The accuracy of correctly identifying pure pigment data of the unmix algorithm is *acc*_test_ = (83 ± 0), that of the unmix-nobb algorithm is *acc*_test_ = (88 ± 0), and that of the unmix-nolb algorithm is *acc*_test_ = (92 ± 0). A comprehensive summary of unmixing and SVM algorithm accuracies is shown in table S2 in the Supplementary Materials. A graphical scheme of the unmixing algorithm and its data flow is shown in fig. S19. For unmixing of pigment mixtures, the entire pure pigment data are used as average reference TA curves.

#### Support vector machine

An SVM is a supervised learning algorithm that classifies data into one of two classes. The algorithm takes *n*-dimensional input vectors (here TA curves consisting of 24 time delays) and separates them by an *n*−1–dimensional hyperplane. This plane maximizes the margin between classes and is defined by the support vectors, the data points from each class that are nearest to the hyperplane and most influence its position. An SVM can be expanded to multiclass classification with a “one-versus-rest” strategy. It naturally lends itself to heterogeneous data and is well suited for high-dimensional data. We use the scikit-learn 1.4 (*58*) and the imbalanced-learn 0.12.0 (*59*) Python packages for training, validation, and testing of the SVM. We randomly select around 27,500 single-pixel P-P TA curves (imblearn RandomUnderSampler), 5500 from each reference sample. We split them into training and testing sets with a ratio of 3:1. We perform a hyperparameter optimization (scikit-learn GridSearchCV) of *C* and γ for the SVM (scikit-learn SVC with radial basis functions as the kernel). The regularization parameter *C* controls the trade-off between minimizing error on the training data and maintaining a smooth decision boundary. Lower values of *C* encourage a simpler, smoother decision boundary, which may allow for some misclassification in the training set but is more likely to generalize well to the test data. In contrast, higher values of *C* prioritize the correct classification of all training data, resulting in a more complex decision boundary that often leads to overfitting. The γ parameter determines the influence of a single training example on the decision boundary. A large γ value confines the influence of a training example to its immediate neighbors, which can create a more complex model that may overfit the data. Conversely, a smaller γ value allows each training example to have a broader influence, leading to a smoother decision boundary that is more likely to generalize better to unseen data. The hyperparameter optimization is performed on the training set with a stratified fivefold cross-validation strategy (scikit-learn StratifiedKFold), with accuracy as the scoring metric, and with a standard scaler applied to all TA curves (scikit-learn StandardScaler). The performance of the best classifier is inferred by measuring accuracy of the classifier applied on the test set. Accuracy is defined as the percentage of correctly classified pixels divided by all classified pixels. The entire procedure is repeated five times, and we compute the average and SD over all five runs.

We train three SVM classifiers: SVM, which is trained on all pigments (bone black, charcoal, graphite, lamp black, and ultramarine blue); SVM-nobb, which is trained without the pigment bone black; and SVM-nolb, which is trained without the pigment lamp black. The validation accuracy for the SVM that is trained on all pigments is *acc*_valid_ = (85.15 ± 0.15)%, and the testing accuracy is *acc*_test_ = (85.43 ± 0.34)%. For the classifier SVM-nobb, the validation accuracy is *acc*_valid_ = (95.88 ± 0.04)% and the testing accuracy is *acc*_test_ = (96.01 ± 0.14)%, and for the classifier SVM-nolb, the validation accuracy is *acc*_valid_ = (94.57 ± 0.11)% and the testing accuracy is *acc*_test_ = (94.40 ± 0.38)%. The accuracies based on the validation set are comparable to the accuracies based on the test set for all three classifiers, which lets us conclude that they are well trained and that we capture the whole range of signal variety. We then use the optimal hyperparameters and the entire reference data to train the final SVM classifiers that are used to classify two-pigment mixtures. Note that the final classifiers are solely trained on reference pigment data and have not been trained with any data from mixed samples. The confusion matrix of each classifier is shown in fig. S20. A comprehensive summary of unmixing and SVM algorithm accuracies is shown in table S2 in the Supplementary Materials. A graphical scheme of the SVM algorithm and its data flow is shown in fig. S21.
