# Supplementary material for: Noninvasive identification of carbon-based black pigments with pump-probe microscopy
Source: Sci Adv. 2024 Dec 11;10(50):eadp0005. doi: 10.1126/sciadv.adp0005 (PMC11633755; doi:10.1126/sciadv.adp0005)
Supplement: Supplementary file 1 — Figs. S1 to S21 Tables S1 to S4 [file sciadv.adp0005_sm.pdf]

Science Advances
AAAS

Supplementary Materials for

Noninvasive identification of carbon-based black pigments with pump-probe microscopy

Heidi V. Kastenholz *et al.*

Corresponding author: Martin C. Fischer, martin.fischer@duke.edu

Sci. Adv. **10**, eadp0005 (2024)
DOI: 10.1126/sciadv.adp0005

This PDF file includes:

Figs. S1 to S21
Tables S1 to S4

Supplementary Materials

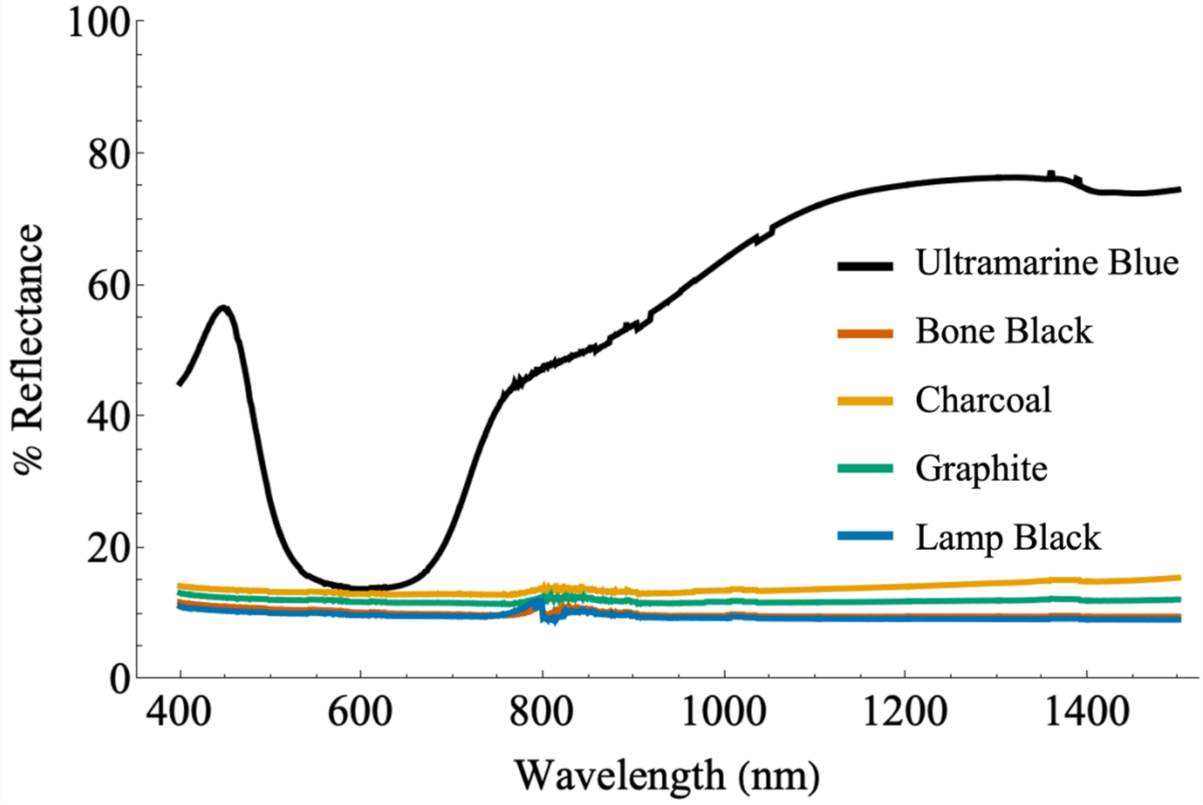

Fig. S1.

Reflectance spectra. The reflectance spectra of the four carbon-based black pigments and ultramarine blue. There was a change in the detector used at 800 nm, which resulted in a percent Reflectance shift, more noticeable for some pigments than others, at this wavelength.

Pigment	Element 1	Element 2	Element 3	Element 4	Element 5	Element 6	Element 7
Bone Black	C: 44.1%	O: 29.4%	Ca: 16.8%	P: 6.9%	Cu: 1.5%		
Charcoal	C: 84.0%	O: 14.5%					
Graphite	C: 84.8%	O: 10.5%	Si: 1.9%	Al: 1.0%			
Lamp Black	C: 94.3%	O: 4.4%					
Ultramarine Blue	O: 41.9%	Si: 18.0%	Na: 11.2%	Al: 9.8%	C: 8.9%	S: 7.9%	Cu: 1.6%

Table S1.

Summary of elements identified in pigments. For clarity, only elements that were identified to be more than or equal to 1.0% of the weight were included. Note that copper (Cu) signals originate from the copper tape used for sample mounting.

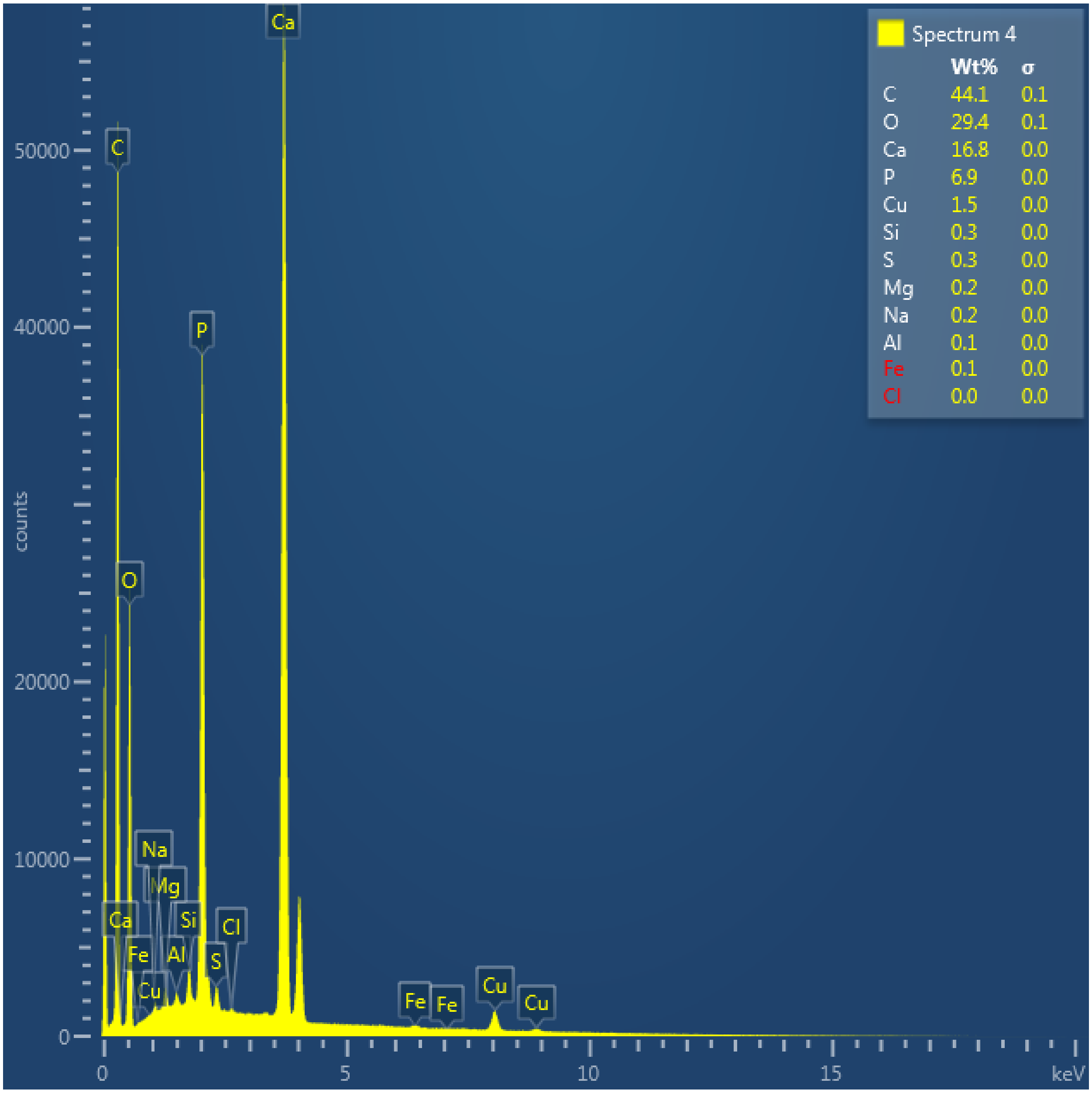

Fig. S2.
Elemental Spectrum for Bone Black.

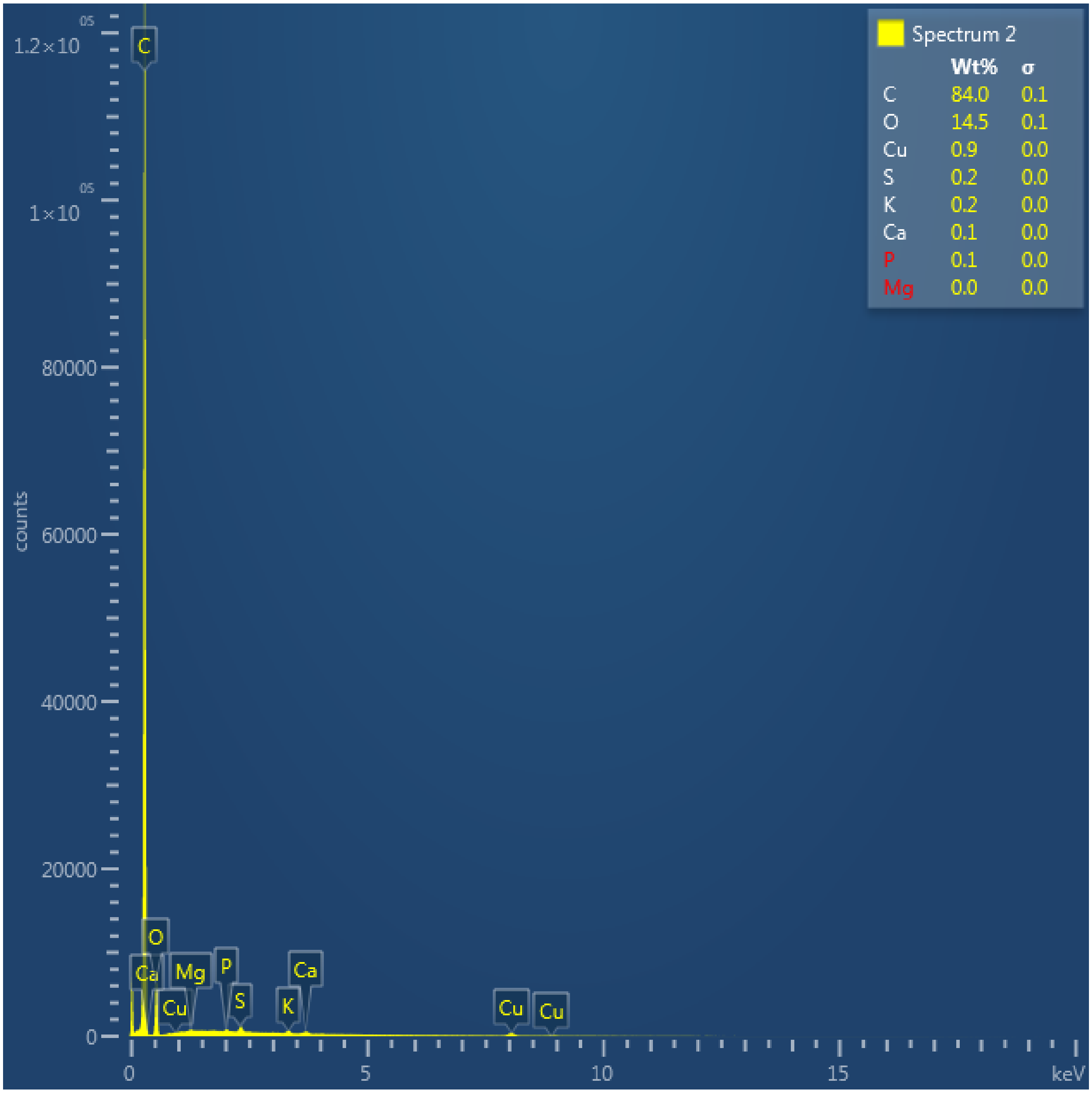

Fig. S3.

Elemental Spectrum for Charcoal.

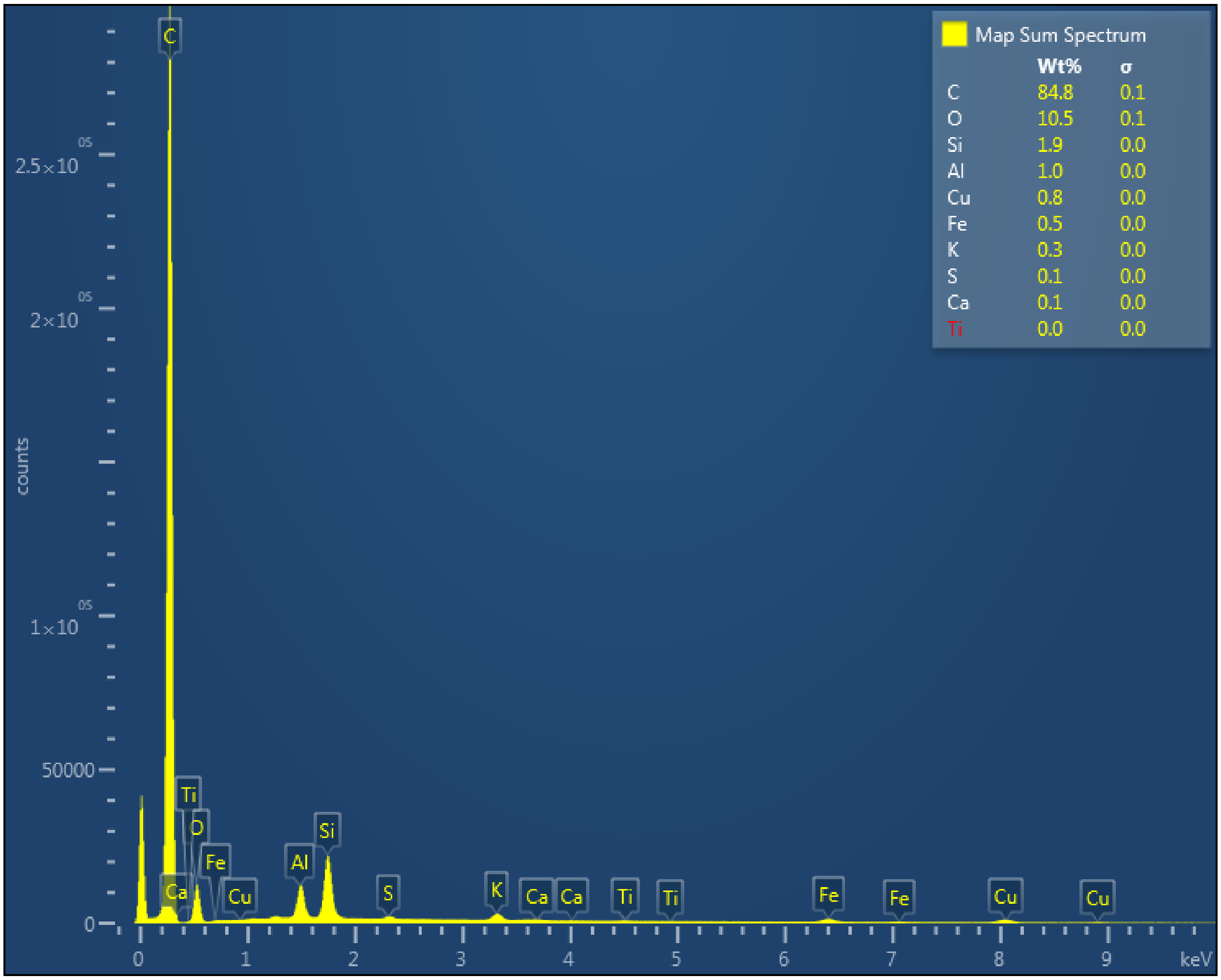

Fig. S4.
Elemental Spectrum for Graphite.

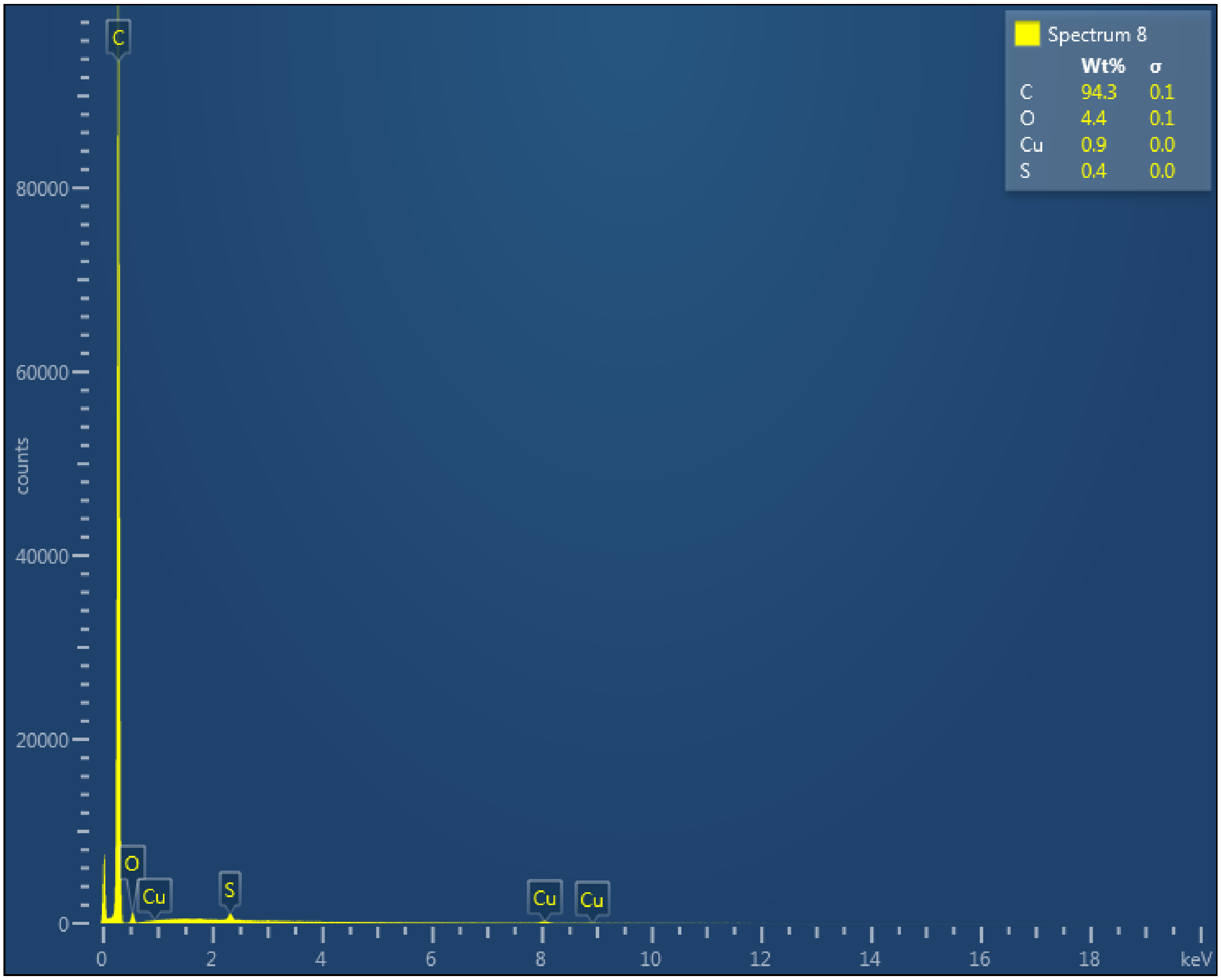

Fig. S5.

Elemental Spectrum for Lamp Black.

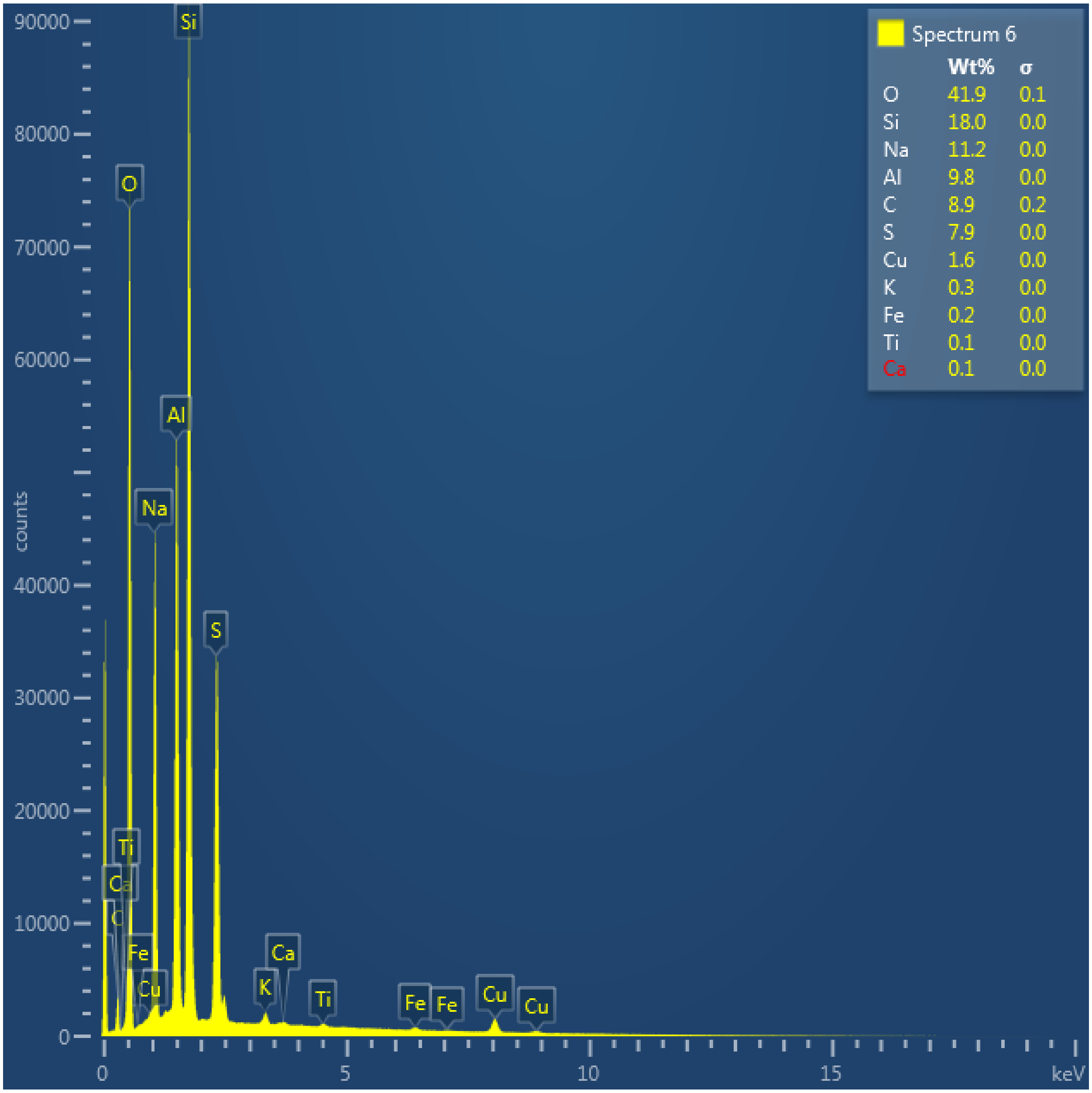

Fig. S6.
Elemental Spectrum for Ultramarine Blue.

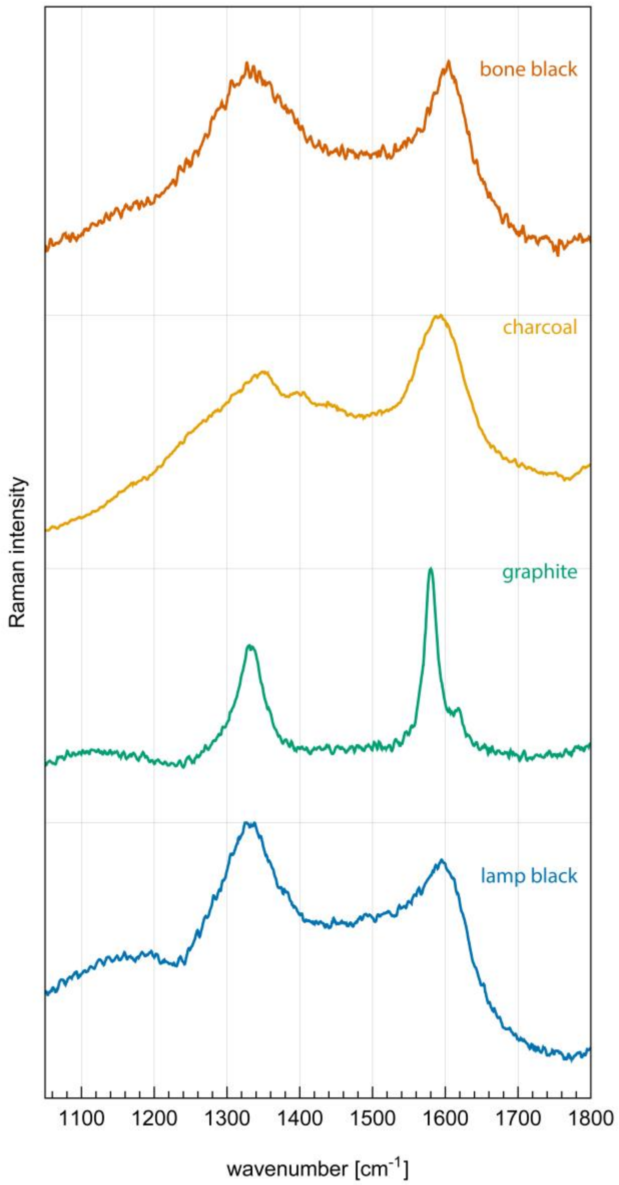

Fig. S7. Raman spectra of pure carbon-based black pigments

The presence of the two peaks at approximately 1350 and 1590 cm^{-1} confirm the pigments as carbon-based black pigments with varying levels of disorder. Graphite, being the most ordered pigment, has the sharpest peaks. As the other three have more molecular disorder present, the G and D bands are broader and are expected to be sample dependent.

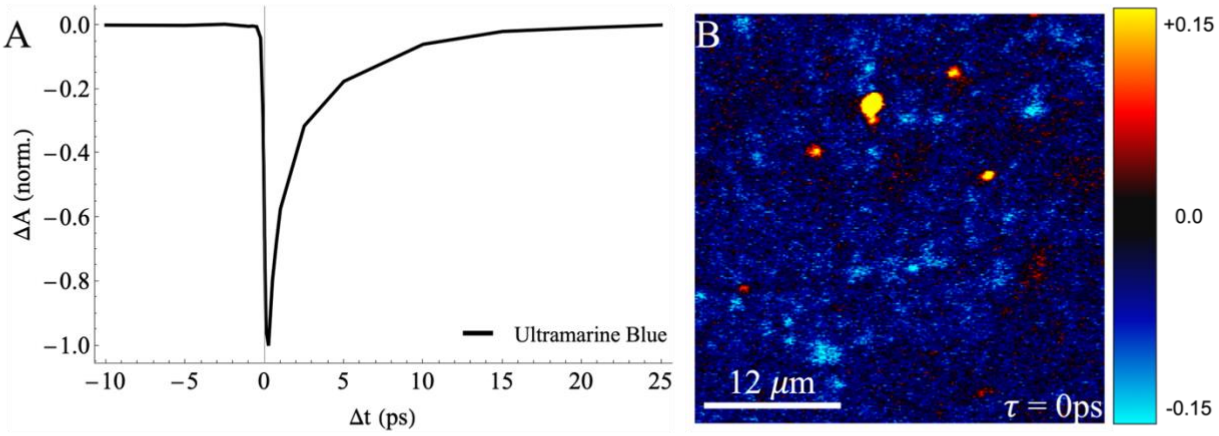

Fig. S8.

Average transient absorption curve and pump-probe image for ultramarine blue. An average transient absorption curve of ultramarine blue, corresponding to the region of interest (36 µm x 36 µm) shown in part B at a time overlap Δt = 0 ps.

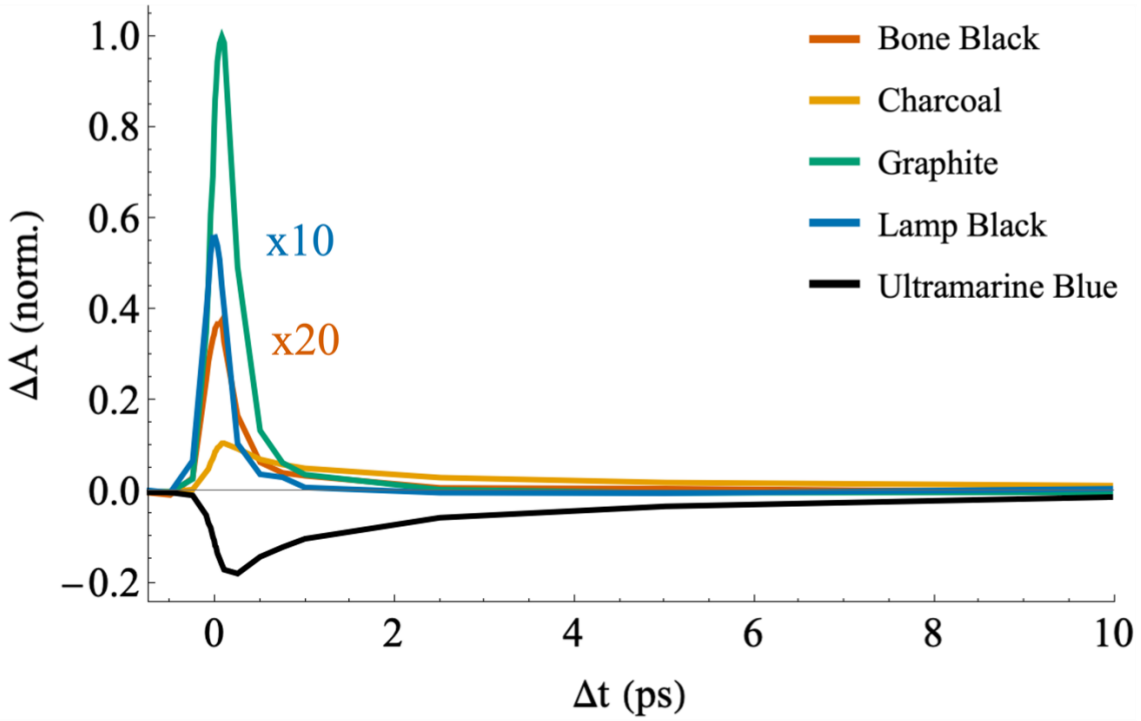

Fig. S9.
Transient absorption curves of bone black, charcoal, graphite, lamp black, and ultramarine blue. Each curve was normalized to the Graphite maximum, and then scaled (bone black x20, charcoal x1, graphite x1, lamp black x10, ultramarine blue x1) for visual comparison.

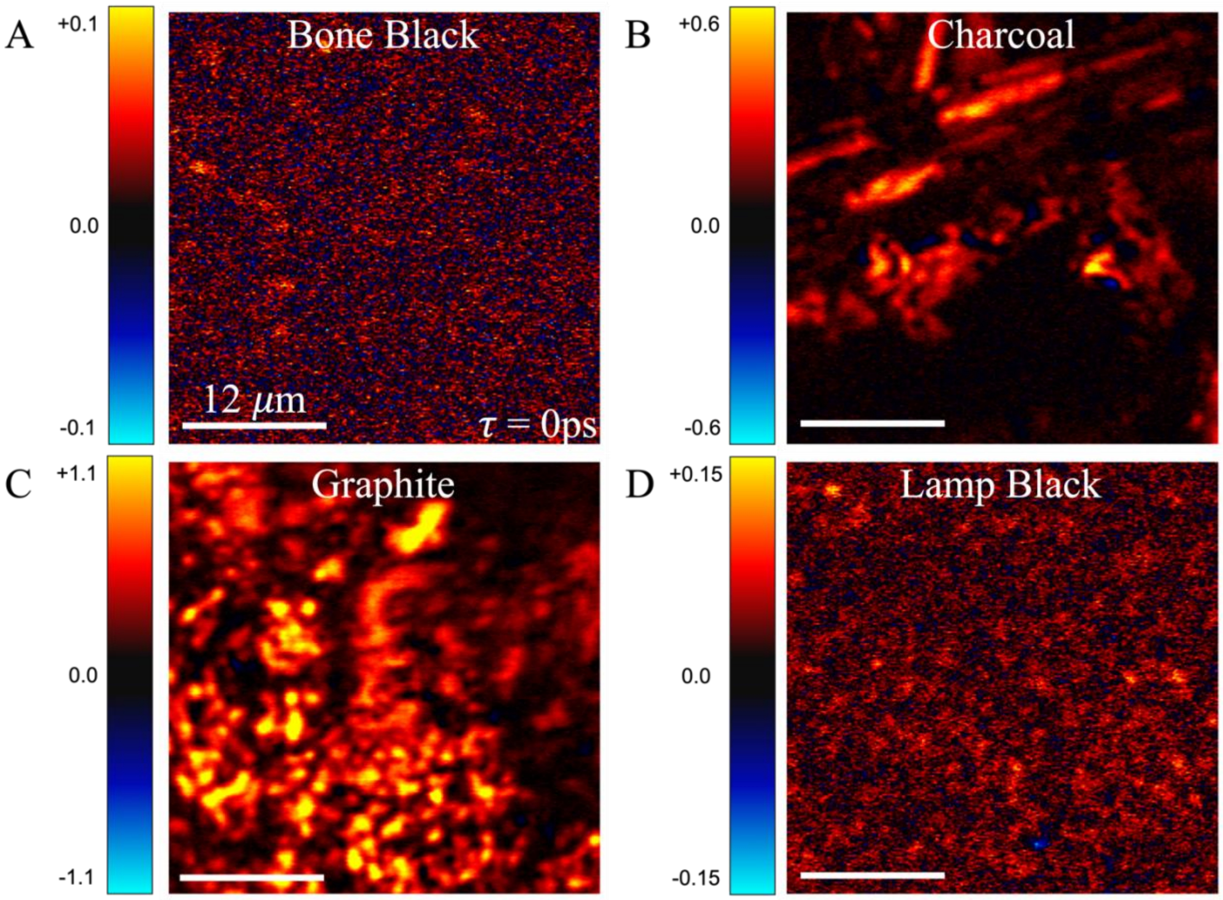

Fig. S10.
Pump-probe images for bone black, charcoal, graphite, and lamp black. Each image represents the pump-probe image at a time delay of Δt=0ps of an area of 36μm x 36μm for A) Bone Black B) Charcoal C) Graphite D) Lamp Black. Note the different false-coloring scales for each image.

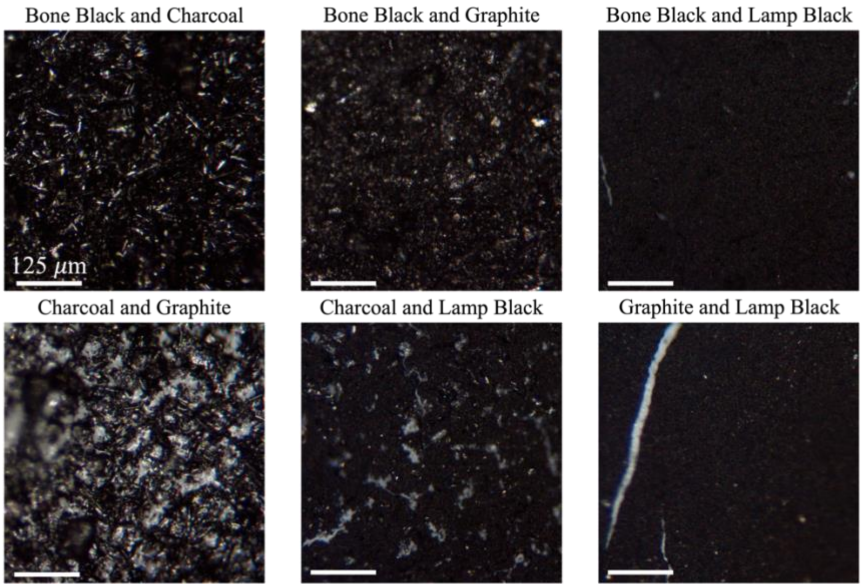

Fig. S11.

Brightfield images of the six black-black mixtures. Each image is a 500 x 500 micrometer field of view, the scale bar represents 125 micrometers.

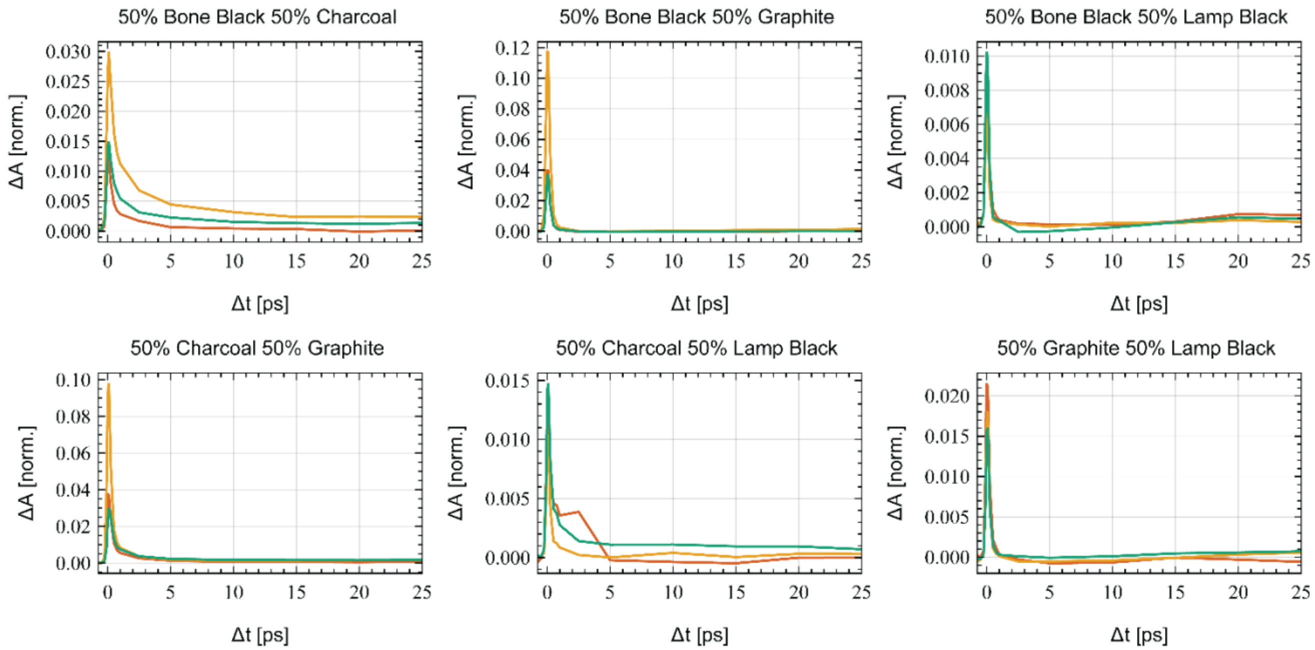

Fig. S12.

Average transient absorption curves of all black-black mixtures. The three colors represent three different regions of interest.

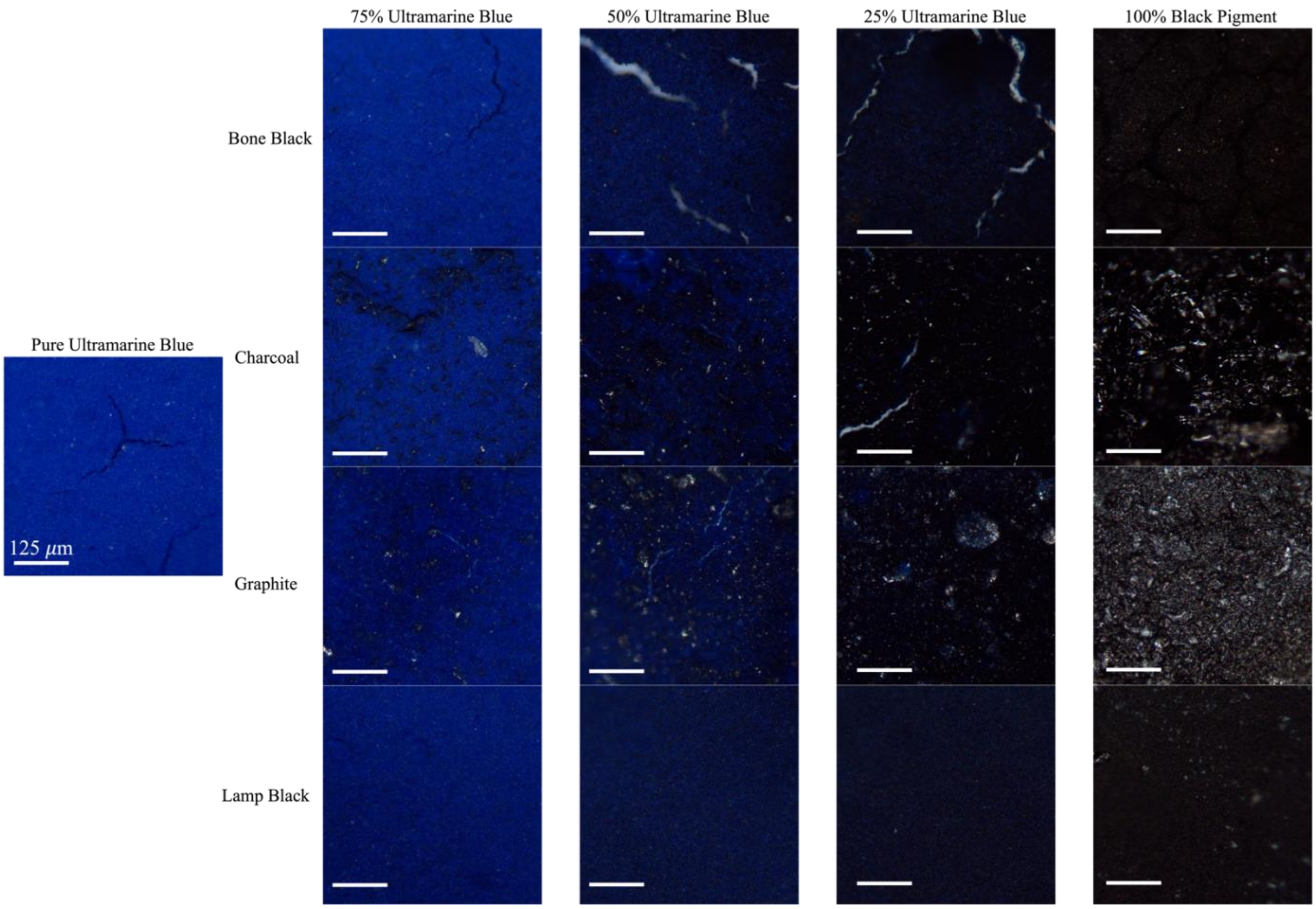

Fig. S13.

Brightfield images of the twelve shading mixtures and the five pure pigments. The standalone leftmost image is of pure ultramarine blue. The columns going from left to right the images are decreasing amounts of ultramarine blue, 75%, 50%, 25%, 0%, with the last column being pure black pigment. The rows, from top to bottom, are bone black, charcoal, graphite, and lamp black. Each image is a 500 x 500 micrometer field of view, the scale bar represents 125 micrometers.

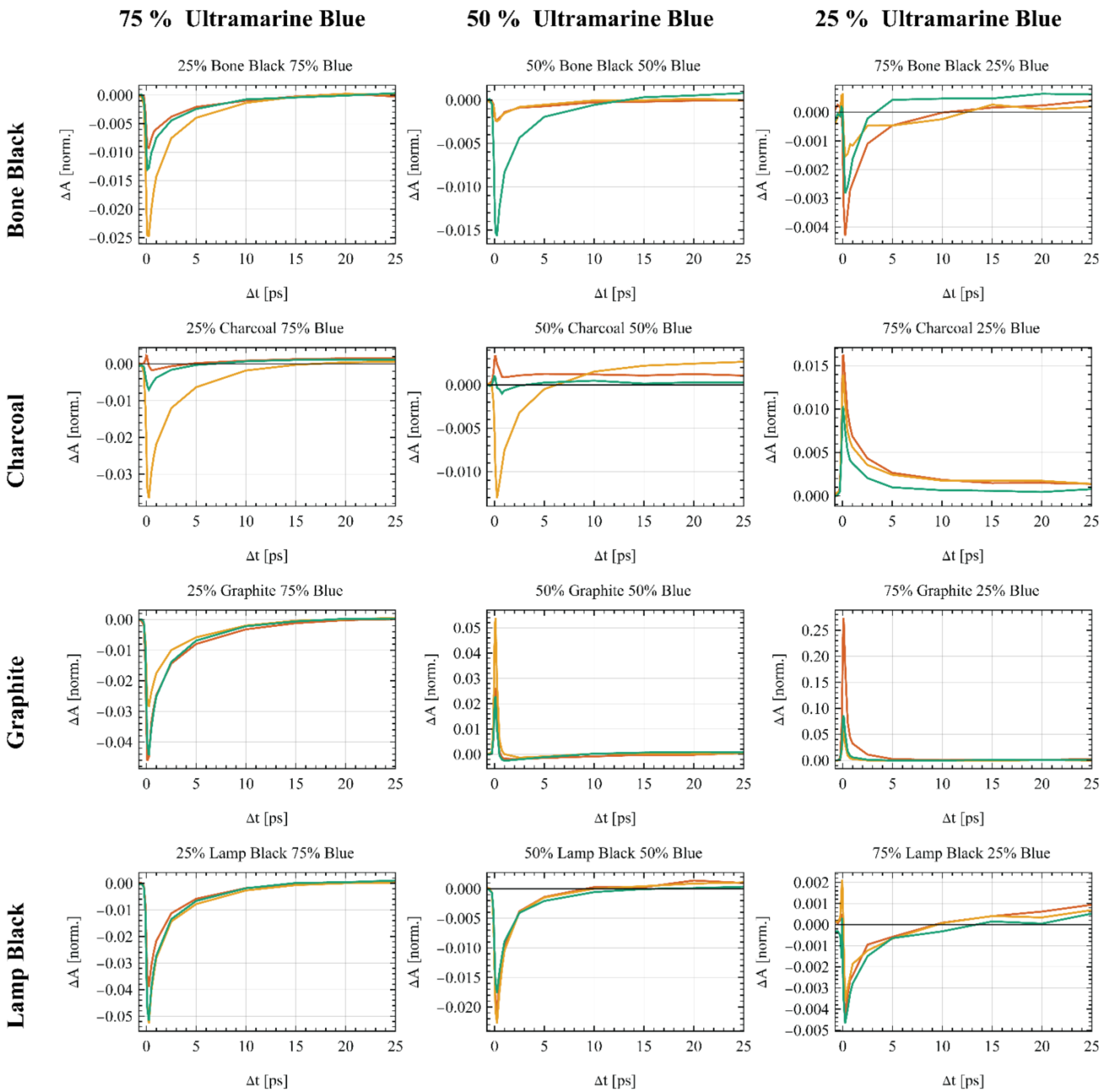

Fig. S14.

Average transient absorption curves of all blue-black mixtures. The three colors represent three different regions of interest.

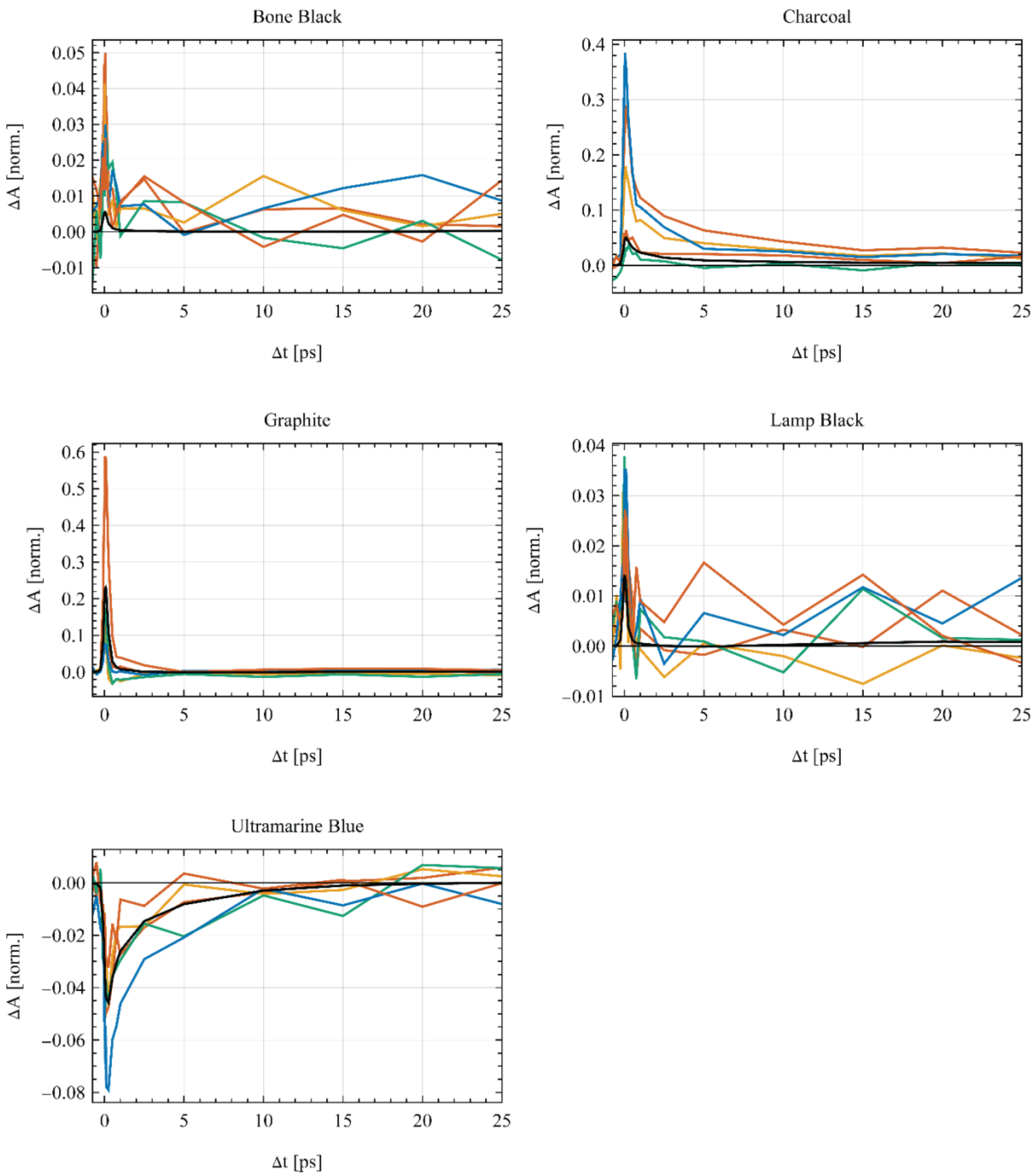

Fig. S15.

Randomly selected single pixel transient absorption curves of all five pigments. Each plot contains 5 colored, randomly selected, single pixel transient absorption curves of the respective pure pigment for comparison. The average transient absorption curves based on all pixel in the field of view per pigment are shown in black.

Classifier	Average Accuracy Pure Samples* [%]	Average Accuracy Black-Black Mixtures [%]	Average Accuracy Black-Blue Mixtures [%]
SVM	85	74	82
SVM-nobb	96	90	84
SVM-nolb	94	85	88
Unmixing	83	63	62
Unmixing-nobb	88	76	72
Unmixing-nolb	92	69	64

Table S2.
Overall accuracy for SVM, SVM-nobb, SVM-nolb, unmixing, unmixing-nobb, and unmixing-nolb classifiers. All classifiers are solely trained on pure pigment data. *To infer classifier accuracy for pure pigment data, pure pigment data was randomly split into train and test set. Train data was used for training and hyperparameter optimization (SVM) or to compute reference curves (unmixing). Accuracy was measured with test data.

Mixture	Overall [%]			Bone Black [%]			Charcoal [%]			Graphite [%]			Lamp Black [%]			Ultramarine Blue[%]		
	SVM	SVM No BB	SVM No LB	SVM	SVM No BB	SVM No LB	SVM	SVM No BB	SVM No LB	SVM	SVM No BB	SVM No LB	SVM	SVM No BB	SVM No LB	SVM	SVM No BB	SVM No LB
Bone – Char	93±1	-	96±1	28±9	-	30±10	65±9	-	66±9	2±1	-	2±1	3±2	-	-	2±2	-	2±2
Bone – Grap	61±2	-	79±6	36±3	-	49±3	19±2	-	21±6	25±4	-	29±6	20±2	-	-	1±0	-	1±0
Bone – Lamp	97±1	-	-	62±7	-	-	2±1	-	-	1±0	-	-	35±8	-	-	0±0	-	-
Char – Grap	79±3	86±2	81±4	11±1	-	13±1	34±8	36±7	36±8	45±11	50±9	45±11	4±1	8±2	-	5±2	6±3	5±3
Char – Lamp	55±7	94±5	-	40±8	-	-	30±9	37±12	-	5±3	6±4	-	25±9	56±10	-	1±1	1±1	-
Grap – Lamp	57±2	89±4	-	37±2	-	-	6±2	10±4	-	24±5	27±6	-	34±5	63±7	-	0±0	0±0	-

Table S3.

Support Vector Machine classification accuracy for black-black mixtures. Abbreviations: Bone Black: Bone/BB; Charcoal: Char; Graphite: Grap; Lamp Black: Lamp/LB; Support Vector Machine: SVM. Note that percentages have been rounded to integers and therefore might not add to 100%. Accuracies are averaged over multiple regions of interest with their standard deviations as error margins.

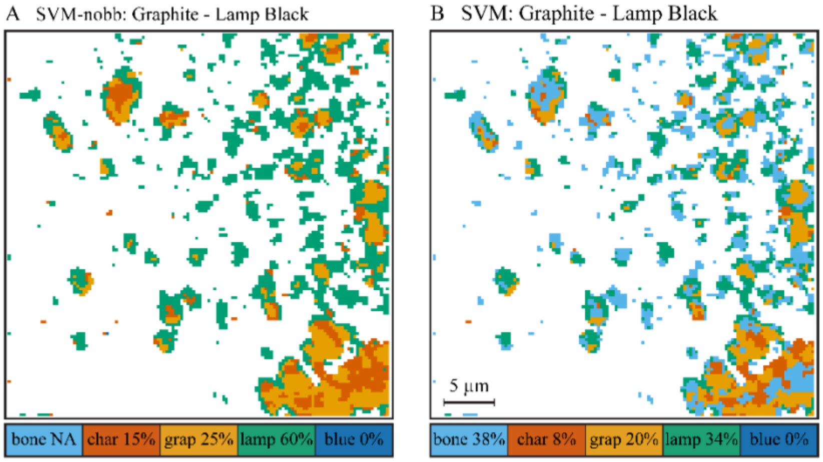

Fig. S16.

Pigment map comparison between SVM-nobb and SVM of Graphite – Lamp Black. A. The pigment map of Graphite – Lamp Black derived with the SVM-nobb algorithm. B. The pigment map for the same region derived with the SVM algorithm. The percentages are for the specific field of view shown here and might deviate from the averages. Abbreviations: Bone Black: Bone; Charcoal: Char; Graphite: Grap; Lamp Black: Lamp; Support Vector Machine: SVM; NA: not applicable.

Mixture	Overall [%]			Bone Black [%]			Charcoal [%]			Graphite [%]			Lamp Black [%]			Ultramarine Blue[%]		
	SVM	SVM No BB	SVM No LB	SVM	SVM No BB	SVM No LB	SVM	SVM No BB	SVM No LB	SVM	SVM No BB	SVM No LB	SVM	SVM No BB	SVM No LB	SVM	SVM No BB	SVM No LB
75% Blue – 25% Bone	95±1	-	93±1	12±10	-	12±10	4±1	-	6±1	1±0	-	1±0	0±0	-	-	83±11	-	81±10
50% Blue – 50% Bone	78±15	-	74±18	24±16	-	24±14	19±14	-	25±16	1±0	-	2±2	2±1	-	-	54±30	-	50±30
25% Blue – 75% Bone	75±4	-	79±5	37±3	-	44±2	14±5	-	17±7	1±1	-	3±2	10±4	-	-	39±5	-	36±7
75% Blue – 25% Char	96±3	98±1	96±3	3±3	-	4±3	24±19	25±20	25±19	0±0	0±0	1±0	0±0	1±1	-	72±21	74±21	71±22
50% Blue – 50% Char	83±13	93±5	83±12	14±12	-	16±12	47±26	54±32	48±26	1±0	1±1	1±0	2±1	6±5	-	40±40	40±40	40±40
25% Blue – 75% Char	79±6	89±4	78±6	18±5	-	19±6	77±5	87±3	77±5	2±1	3±1	2±1	2±0	9±3	-	2±1	2±1	2±1
75% Blue – 25% Grap	93±3	96±2	92±3	1±1	-	1±1	6±2	4±2	6±2	5±1	5±2	5±1	0±0	1±0	-	88±3	90±2	87±3
50% Blue – 50% Grap	77±3	83±2	78±2	8±1	-	10±1	10±1	11±0	12±1	52±5	55±5	54±5	5±0	7±1	-	25±4	28±5	24±4
25% Blue – 75% Grap	82±9	85±8	83±8	5±3	-	8±5	7±3	7±3	9±3	78±13	80±12	79±11	6±3	8±4	-	4±4	5±4	4±3
75% Blue – 25% Lamp	97±0	98±0	-	0±0	-	-	1±0	1±0	-	1±0	1±0	-	0±0	0±0	-	97±0	98±0	-
50% Blue – 50% Lamp	82±4	87±3	-	6±2	-	-	9±2	9±2	-	3±1	4±1	-	2±0	2±0	-	80±4	85±3	-
25% Blue – 75% Lamp	50±5	64±4	-	28±2	-	-	21±3	33±4	-	2±0	3±0	-	30±9	39±10	-	20±5	25±8	-

Table S4.

Support Vector Machine classification accuracy for shading mixtures. Abbreviations: Bone Black: Bone/BB; Charcoal: Char; Graphite: Grap; Lamp Black: Lamp/LB; Support Vector Machine: SVM. Note that percentages have been rounded to integers and therefore might not add to 100%. Accuracies are averaged over multiple regions of interest with their standard deviations as error margins.

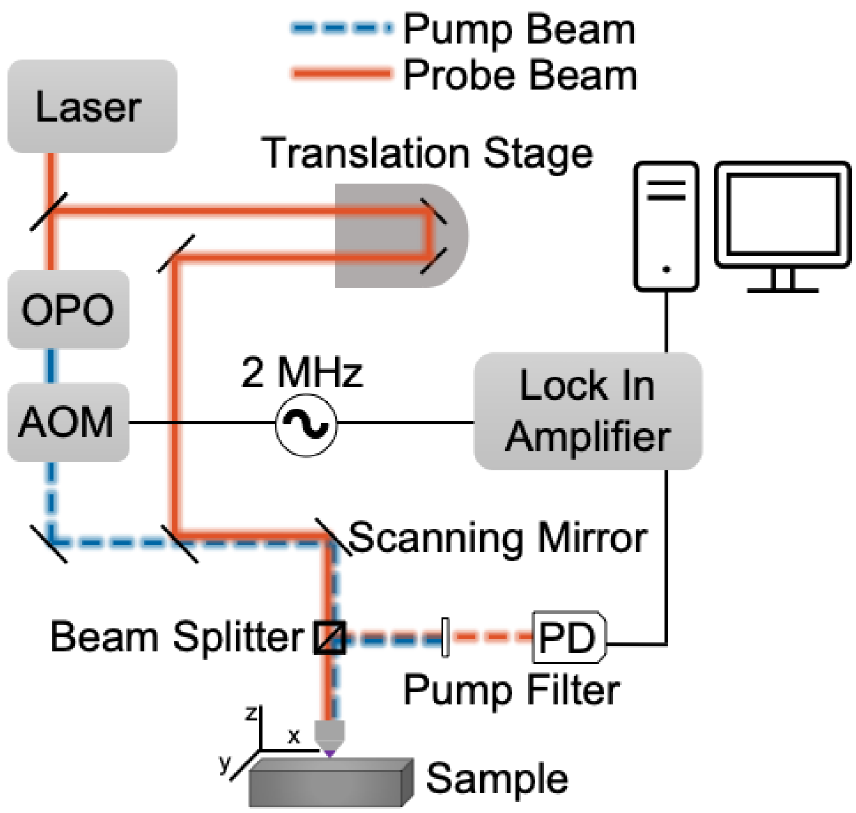

Fig. S17.

Schematic of experimental set-up for pump-probe microscopy. A femtosecond laser at λ_{probe} = 817 nm is split into two spatial modes. One mode serves as pump for an optical parametric oscillator (OPO) creating the pump wavelength at λ_{pump} = 720 nm , which is also amplitude modulated at a frequency of 2 MHz with an acousto-optic modulator (AOM). The pump and probe beams are spatially overlapped and coupled into a conventional laser scanning microscope.

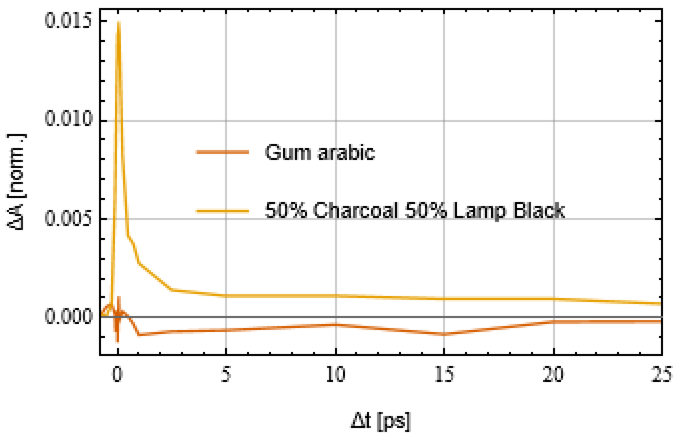

Fig. S18.

Transient absorption curve of gum arabic. The transient absorption curve of pure gum arabic (red) is negligible with respect to transient absorption curves containing pigment, e.g. a 50-50 mixture of charcoal and lamp black (yellow).

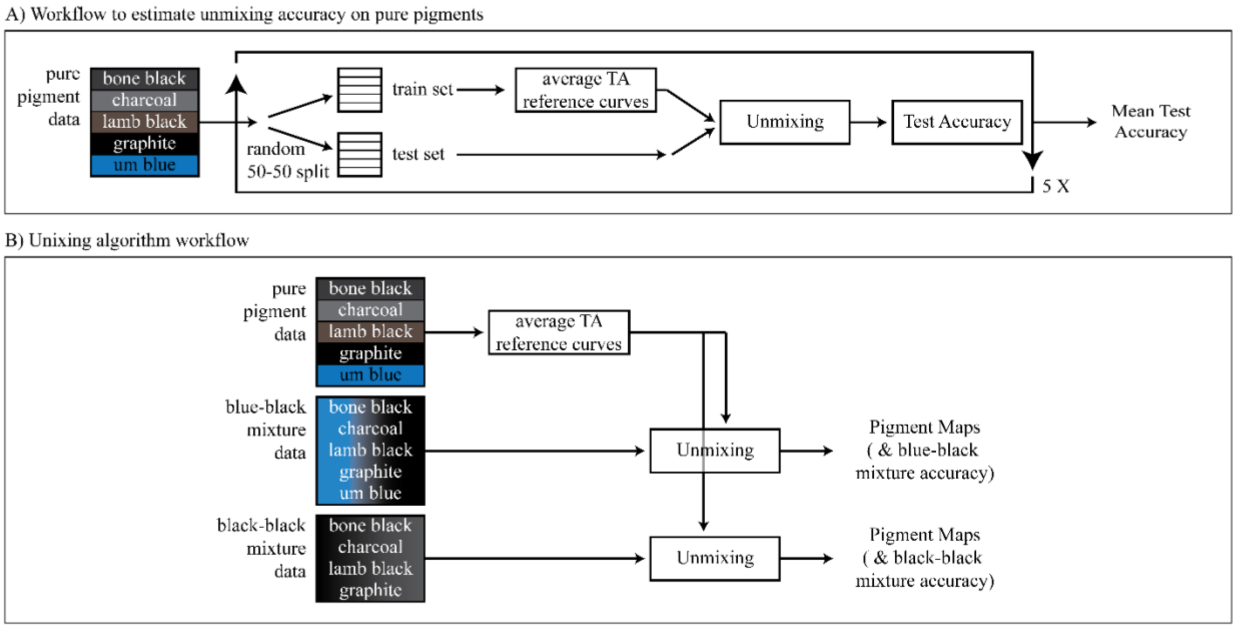

Fig. S19

Unmixing algorithm schematic. A) Workflow to estimate unmixing accuracy on pure pigments. B) Workflow for pigment mixture classification.

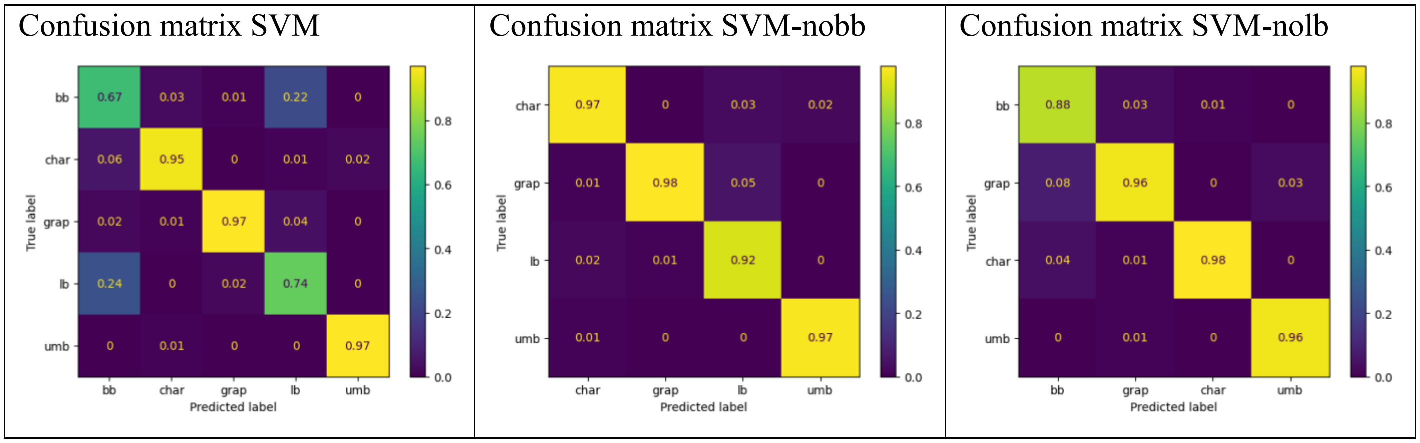

Fig. S20.

Confusion matrix for classifiers SVM, SVM-nobb, and SVM-nolb for pure pigments.

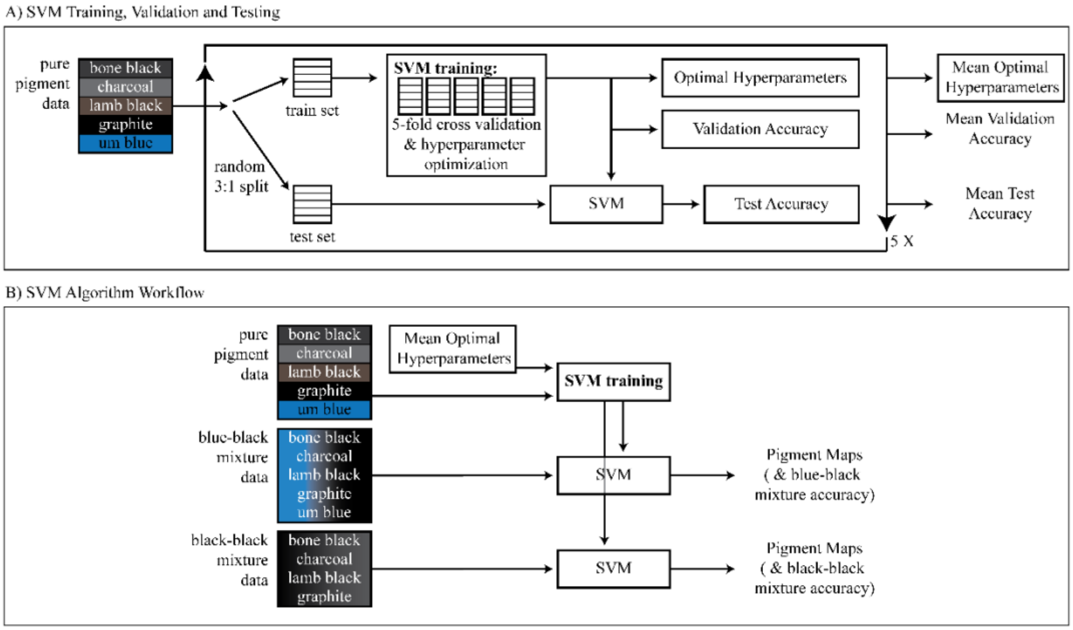

Fig. S21. Support Vector Machine algorithm schematic. A) Workflow to estimate SVM validation accuracy, testing accuracy and optimal hyperparameter on pure pigments. B) Workflow for pigment mixture classification.
